# Click-Capable Phenanthriplatin Derivatives as Tools to Study Pt(II)-Induced Nucleolar Stress

**DOI:** 10.1021/acschembio.3c00607

**Published:** 2024-03-14

**Authors:** Paul D. O’Dowd, Andres S. Guerrero, Katelyn R. Alley, Hannah C. Pigg, Fiona O’Neill, Justine Meiller, Chloe Hobbs, Daniel A. Rodrigues, Brendan Twamley, Finbarr O’Sullivan, Victoria J. DeRose, Darren M. Griffith

**Affiliations:** † Department of Chemistry, Royal College of Surgeons in Ireland, Dublin D02 YN77, Ireland; ‡ SSPC, The Science Foundation Ireland Research Centre for Pharmaceuticals, Limerick V94 T9PX, Ireland; § Department of Chemistry and Biochemistry, University of Oregon, Eugene, Oregon 97403, United States; ∥ Life Science Institute, Dublin City University, Dublin D09 V209, Ireland; ⊥ Department of Chemistry, Trinity College Dublin, Dublin D02 PN40, Ireland

## Abstract

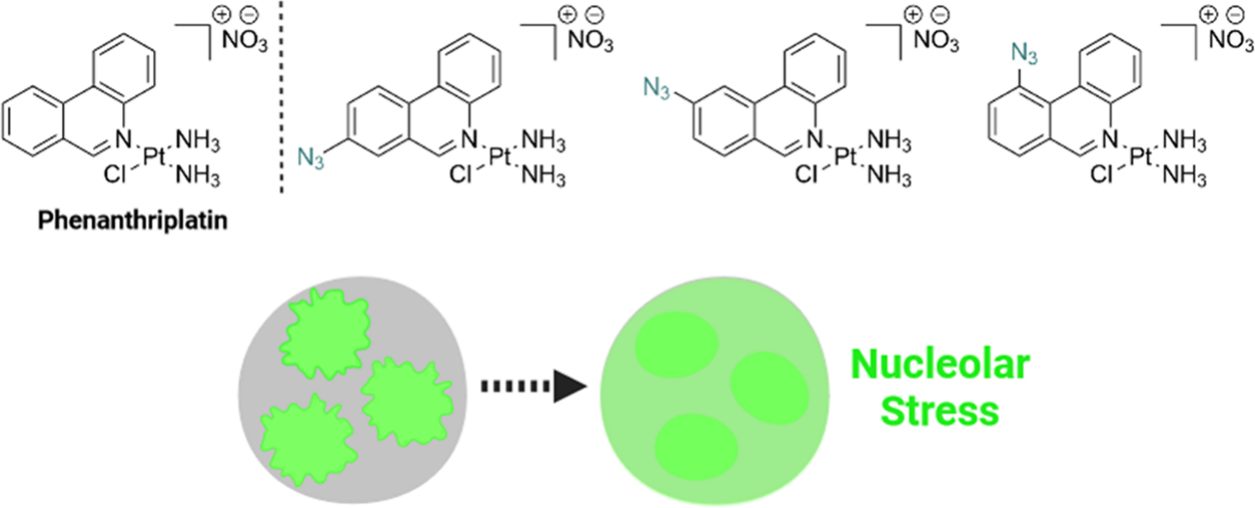

It is well established that oxaliplatin, one of the three Pt(II) anticancer drugs approved worldwide, and phenanthriplatin, an important preclinical monofunctional Pt(II) anticancer drug, possess a different mode of action from that of cisplatin and carboplatin, namely, the induction of nucleolar stress. The exact mechanisms that lead to Pt-induced nucleolar stress are, however, still poorly understood. As such, studies aimed at better understanding the biological targets of both oxaliplatin and phenanthriplatin are urgently needed to expand our understanding of Pt-induced nucleolar stress and guide the future design of Pt chemotherapeutics. One approach that has seen great success in the past is the use of Pt-click complexes to study the biological targets of Pt drugs. Herein, we report the synthesis and characterization of the first examples of click-capable phenanthriplatin complexes. Furthermore, through monitoring the relocalization of nucleolar proteins, RNA transcription levels, and DNA damage repair biomarker γH2AX, and by investigating their *in vitro* cytotoxicity, we show that these complexes successfully mimic the cellular responses observed for phenanthriplatin treatment in the same experiments. The click-capable phenanthriplatin derivatives described here expand the existing library of Pt-click complexes. Significantly they are suitable for studying nucleolar stress mechanisms and further elucidating the biological targets of Pt complexes.

## Introduction

Cisplatin, carboplatin, and oxaliplatin are the only three platinum (Pt) complexes approved for treating cancer worldwide. Together these drugs play an important role in cancer treatment, with central roles in the treatment of testicular, ovarian, bladder, and colorectal cancers.^1,2^ Despite this, the clinical use of Pt agents is commonly hindered by toxic side effects and the development of drug resistance. As such, much research has focused on the design of novel Pt(II) complexes with improved activity, reduced adverse effects, and an ability to overcome Pt-resistance mechanisms.^3−5^ One such complex designed under this premise is phenanthriplatin.^6^

Phenanthriplatin is a monofunctional cisplatin derivative that has increased cellular uptake and has been shown to be 7–40 times more active than cisplatin against a variety of cancer cell lines. Moreover, phenanthriplatin’s spectrum of activity is different from that of the approved Pt chemotherapeutics.^6,7^ Unlike FDA-approved Pt drugs, phenanthriplatin treatment primarily results in the formation of monoadducts with DNA. The formation of these adducts is believed to occur following an initial intercalation step of the phenanthridine ligand between DNA bases, followed by the formation of a Pt-nucleobase DNA monoadduct.^8−11^ Phenanthriplatin-DNA monoadducts have been shown to inhibit RNA polymerase II though they can still be bypassed by DNA polymerase η.^12,13^ Furthermore, phenanthriplatin has also been shown to be an effective topoisomerase II poison.^14^ Taken together, these properties make the design of monofunctional Pt(II) complexes such as phenanthriplatin a promising avenue for overcoming resistance mechanisms associated with clinically approved Pt(II) drugs such as cisplatin and oxaliplatin.^13^

Interestingly, while the DNA-binding properties of phenanthriplatin are well studied, a recent study identified that the primary mechanism of action (MOA) of phenanthriplatin and oxaliplatin is associated with their ability to induce nucleolar stress.^18^ In the same study, the MOA of cisplatin and carboplatin was linked to classical DNA damage response (DDR).^18^ Nucleolar stress is a response pathway that leads to disruption of normal ribosome biogenesis and can ultimately lead to cell apoptosis.^19^ Much research has subsequently been carried out to better understand the nucleolar stress response following oxaliplatin administration.^20−26^ In the case of phenanthriplatin, however, reports remain limited, with the biological pathways activated by the drug still poorly understood.

A previous study has indicated that phenanthriplatin’s capacity to induce nucleolar stress may be dependent on the number of aromatic rings present in the nitrogen donor ligand. Indeed, other monofunctional Pt complexes based on isoquinoline and pyridine, for example, do not induce nucleolar stress responses.^27^ More recently, a monoadduct-generating ruthenium (Ru) complex has also been reported to induce nucleolar stress with a similar biological phenotype to phenanthriplatin.^28^ Given the structural differences between oxaliplatin, phenanthriplatin, and the recently reported octahedral Ru complex, it is clear that nucleolar stress can be initiated despite differences in the nature of ligands and metal centers of anticancer agents. As such, the molecular events leading to nucleolar stress induction may also differ between the complexes. Given these observations and the fact that Pt-induced nucleolar stress is poorly understood, probes to study the molecular targets of phenanthriplatin are of great importance.

**Figure 1:**
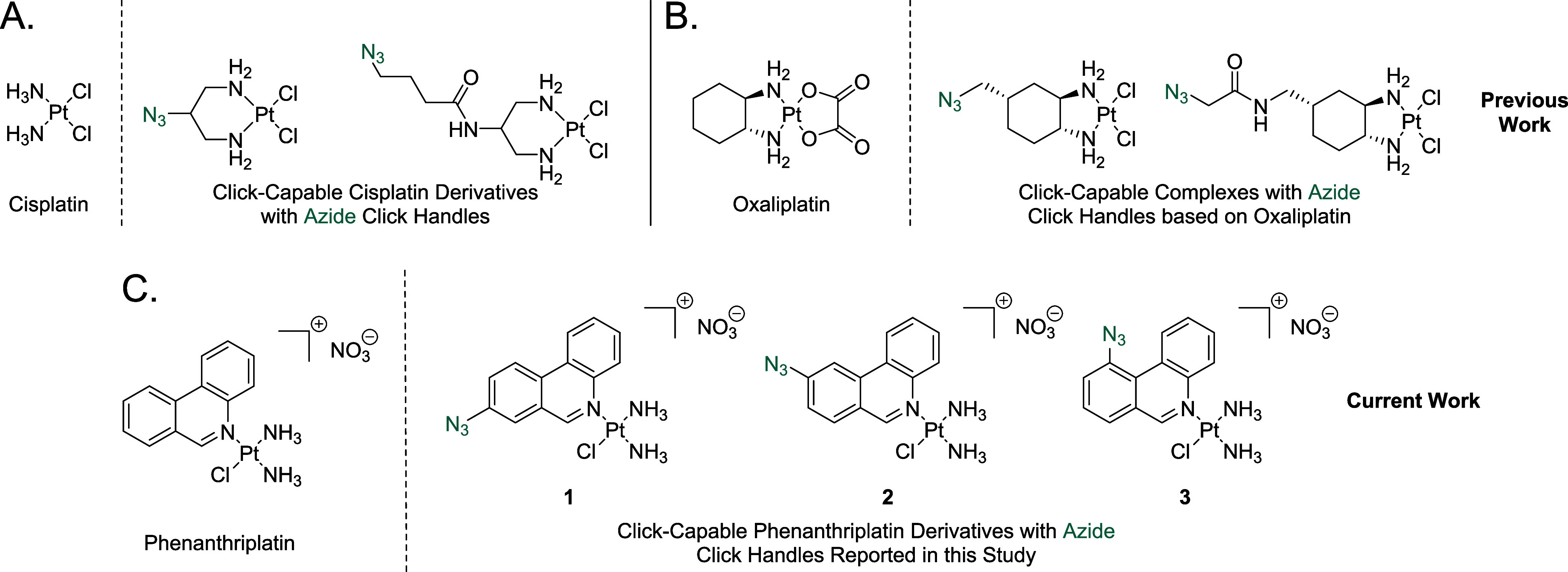
(A) Structures of select previously reported azide-containing cisplatin click derivatives.^15,16^ (B) Structures of previously reported azide-containing oxaliplatin click derivatives.^17^ (C) Structures of click-capable azide-containing phenanthriplatin derivatives reported in this study.

One approach that has previously shown great success in identifying the biological targets of Pt agents is the use of click-capable Pt complexes.^29,30^ The use of cisplatin-like Pt-click complexes for instance has been successfully employed to identify a range of Pt-bound biomolecules such as P(II)-DNA, -RNA, and -protein adducts (Figure 1A).^16,31−35^ Moreover, the design of Pt-click complexes allows for the assembly of more complex molecules such as Pt-click oligonucleotides for target enrichment or Pt-triplex-forming oligonucleotides (TFOs) for gene targeting applications.^36,37^ Recently, our groups reported the first click-capable Pt complexes possessing azide derivatives of oxaliplatin’s 1,2-diaminocyclohexane (DACH) ligand, and demonstrated that one of these complexes could successfully induce nucleolar stress (Figure 1B).^17^ To further expand the range of Pt-click complexes available and to generate a probe that can be used to study potential differences between oxaliplatin- and phenanthriplatin-induced nucleolar stress, we set out to design phenanthriplatin click complexes capable of inducing nucleolar stress responses.

Herein, we report the synthesis and characterization of the first examples of phenanthriplatin click complexes, **1**–**3** (Figure 1C). Through monitoring the redistribution of nucleolar proteins, RNA transcription levels, and biomarkers of DDR, we show that **1**–**3** successfully mimic the biological effects of the parent complex and induces nucleolar stress responses. Furthermore, we show that all of the novel complexes can be functionalized by strain-promoted azide–alkyne click reactions following binding to DNA *in vitro*. Finally, we show that **3** exhibits a cytotoxicity profile similar to that of phenanthriplatin against a range of cancer cell lines. As such, we present **1**–**3** as important tools for studying Pt-induced nucleolar stress alongside recently reported oxaliplatin click complexes.

## Results and Discussion

### Synthesis and Characterization

**Figure 2:**
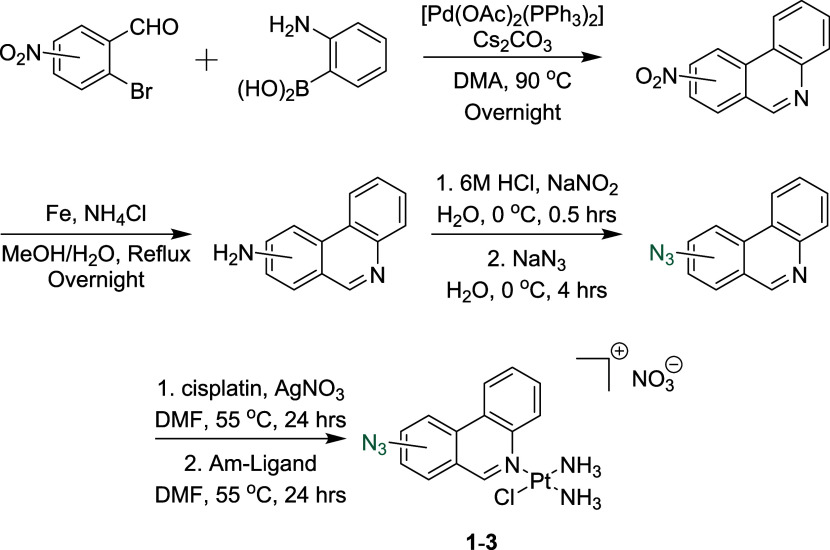
General synthetic pathway for the synthesis of **1**–**3**.

Previous work has shown that the ability of monofunctional platinum complexes to induce nucleolar stress may be dependent on the size of aromatic ring system attached to the nitrogen donor ligand.^27^ Despite this, we hypothesized that small modifications to the phenanthridine ring system of phenanthriplatin would be tolerated, without altering the biological activity of the parent complex. Azide click handles have previously been employed in click-capable Pt(II) complexes as the azide group is a small reactive handle and is highly selective for alkyne click partners via Cu (I)-catalyzed azide–alkyne cycloaddition (CuAAC) and strain-promoted azide–alkyne cycloaddition (SPAAC) reactions.^29^ Given the lack of reports describing the effect of small modifications on the activity of phenanthriplatin, modifications at three different positions on the phenanthridine ring were explored. Synthesis of the novel phenanthridine ligands, incorporating the azide group at the 8-, 9-, and 10-positions, was carried out in three analogous steps from commercially available starting materials (Figure 2). Briefly, an initial Suzuki Reaction coupled with a condensation reaction, led to the formation of the phenanthridine ring system. Subsequent reduction of the nitro functional group and conversion of the resulting amine to an azide via a diazonium salt led to the formation of the desired ligands. Following isolation of the respective ligands, **1**–**3** were synthesized through reaction with cisplatin and silver nitrate in a similar manner to that reported for phenanthriplatin.^6^ The identity and purity of all complexes were subsequently confirmed by NMR and high-resolution mass spectrometry (HRMS) analysis (Figures S3–S11).

**Figure 3:**
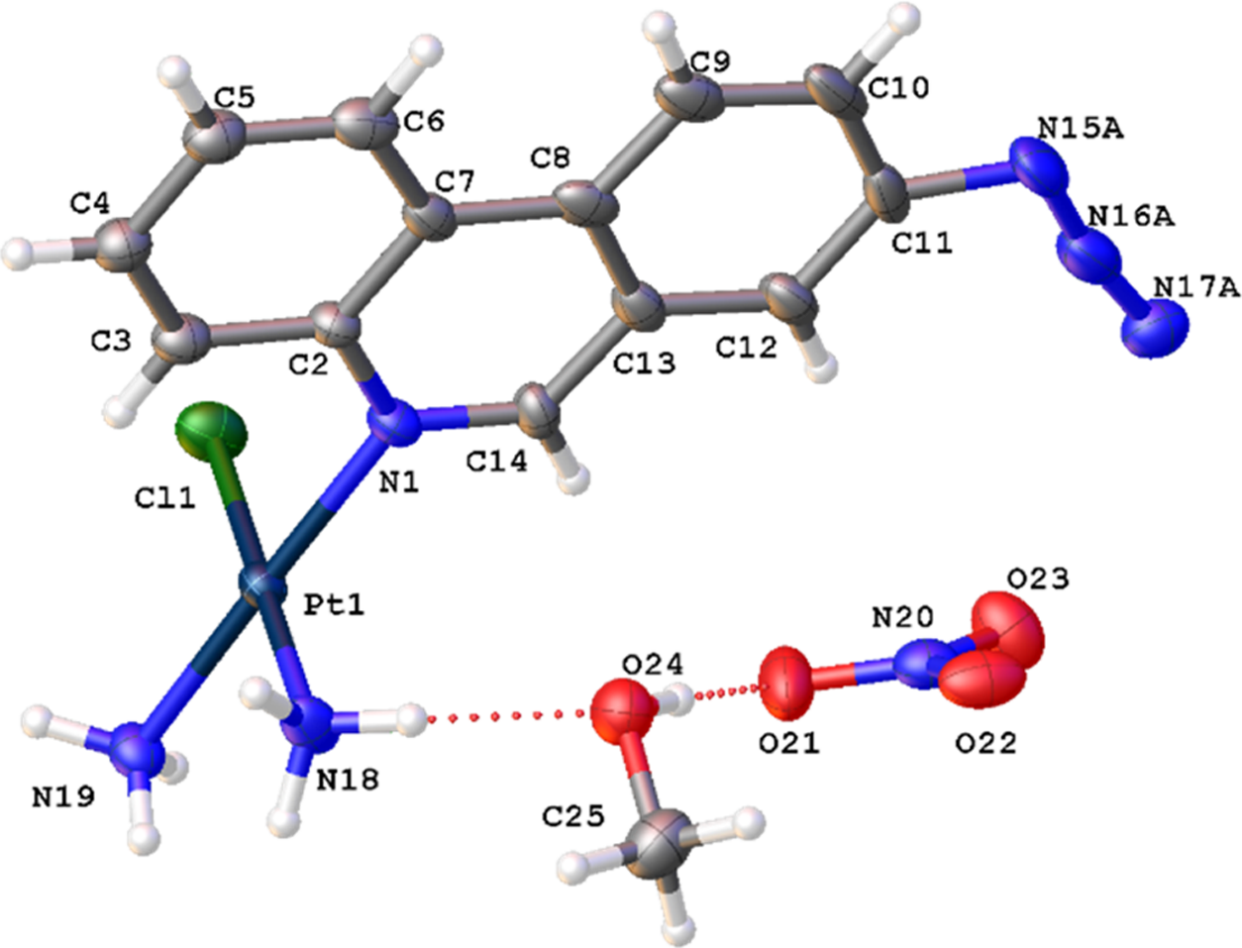
X-ray structure of **1**, showing the majority occupied azide moiety (75%) only. Methanol solvate hydrogen bonds to both NH_3_ and NO_3_^–^ groups. Displacement parameters are shown at 50% probability.

The X-ray crystal structure of **1** was determined, showing successful coordination of the modified phenanthridine ligand to platinum and the desired nitrate counterion (Figure 3). In our case, the complex crystallizes as a methanol solvate (Figure 3). The structure of **1** is similar to the original phenanthriplatin^6^ and displays expected bond lengths and angles (see Table S4). **1** also crystallizes in a centrosymmetric space group, indicating that it is also a racemate. One notable difference is the angle of the plane normal between the phenanthridine and a line joining the pyridyl nitrogen, the Pt atom, and the trans amine nitrogen, 90.92(6)°. In the nitrate salt of phenanthriplatin, this is 68.29(4)°, and in the triflate salt of phenanthriplatin,^38^ 79.15(9)° (see Figure S16). This bending of the phenanthridine moiety, with respect to the coordination plane, has been attributed to packing forces. In **1**, there are significantly more π–π interactions which align the phenanthridine moieties into stacks parallel to the *b*-axis (see Figure S17). As the coordination environment around the metal center has not changed, the steric protection provided by the phenanthridine in 1 is comparable to phenanthriplatin (1, C3–Pt1, 3.184(4) Å; phenanthriplatin, 3.220(8)Å).

### **1**–**3** Induce Nucleolar Protein Redistribution to a Similar Degree as Phenanthriplatin

Following synthesis of **1**–**3**, we turned our attention to investigate whether the novel complexes could induce nucleolar stress in a similar fashion to the parent complex, phenanthriplatin. One hallmark of nucleolar stress is the relocalization of the nucleolar protein nucleophosmin (NPM1). In non-drug-treated cells, NPM1 is found in well-defined regions within the granular region of the nucleolus; however, following induction of nucleolar stress, the protein redistributes throughout the nucleoplasm.^19^

Through immunofluorescence staining techniques, the distribution of NPM1 in A549 cells was quantified by the coefficient of variation (CV, see the Methods section). As previously reported, CV values of ∼0.6 are indicative of cells undergoing nucleolar stress, while CV values ∼1.0 indicate a lack of stress.^21^ Immunofluorescence levels of NPM1 were quantified 24 h following treatment with **1**–**3** at 0.5 μM, as previous studies have indicated that treatment with phenanthriplatin at this concentration results in a strong nucleolar stress response.^27^ Actinomycin D (ActD), an FDA-approved anticancer drug known to induce nucleolar stress, was included in this study alongside known Pt nucleolar stress inducers, oxaliplatin, and phenanthriplatin.^25^

**Figure 4:**
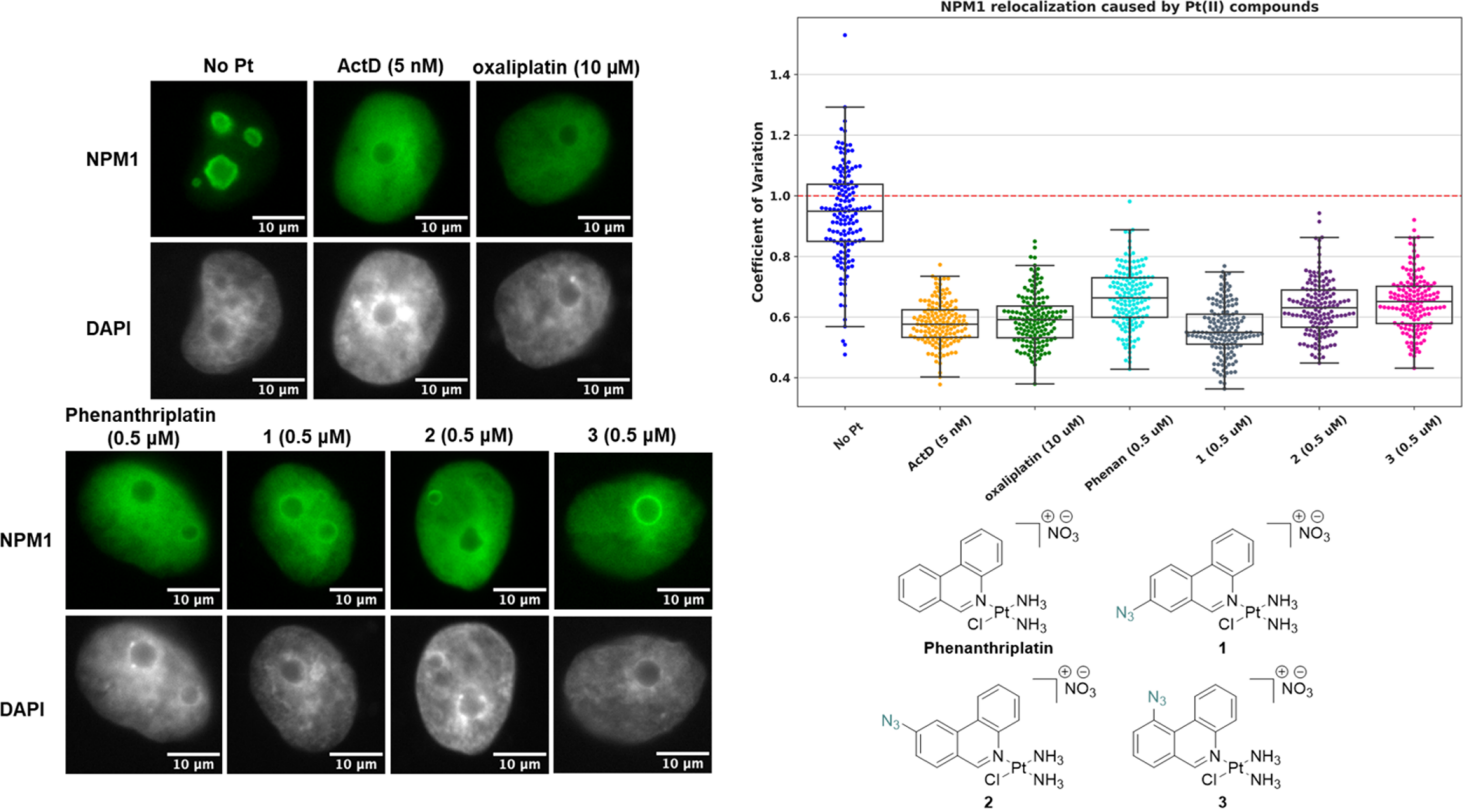
NPM1 relocalization (NPM1: green, 4′,6-diamidino-2-phenylindole, DAPI: gray) induced by **1**–**3** (0.5 μM), phenanthriplatin (0.5 μM), oxaliplatin (10 μM), and ActD (5 nM). Representative images and CV calculations for *n* = 3 provided; see Figure S1 for full cell images. Boxes represent median, first, and third quartiles, where vertical lines are the range of data with outliers (see the Experimental Section). Scale bar = 10 μm.

Following treatment with **1**–**3**, pronounced redistribution of NPM1 throughout the nucleoplasm was observed (Figure 4). Importantly, the degree of redistribution of NPM1 remained similar following treatment with each of **1**–**3** (∼0.6). Furthermore, **1**–**3** were found to induce a similar degree of NPM1 redistribution as the known nucleolar stress inducers ActD, oxaliplatin, and phenanthriplatin.

**Figure 5:**
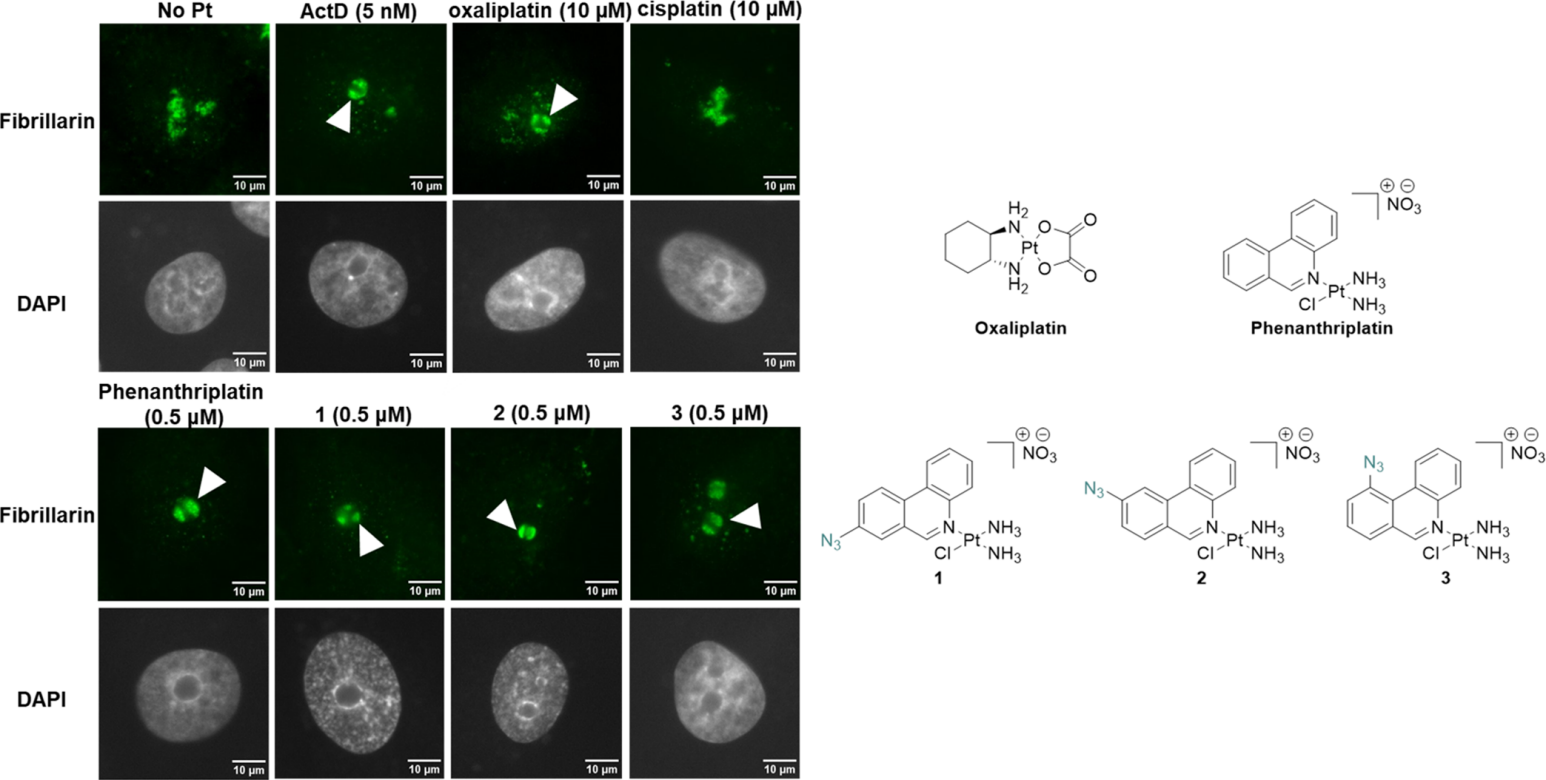
Nucleolar morphological changes monitored by fibrillarin redistribution (fibrillarin: green, DAPI: gray) for ActD (5 nM), oxaliplatin (10 μM), phenanthriplatin (0.5 μM), and **1**–**3** (0.5 μM) at 24 h treatment in A549 cells (*n* = 3). The white arrow indicates nucleolar cap formation. Scale bar = 10 μm.

Redistribution of the nucleolar protein fibrillarin (FBL) is another indicator of nucleolar stress responses.^22,25^ FBL is normally found in the dense fibrillary component of nucleoli; however, upon nucleolar stress, FBL condenses in nucleolar cap-like structures.^39^ Using an FBL immunofluorescence assay, FBL localization in the nucleolus was observed following treatment with ActD, oxaliplatin, and phenanthriplatin. Importantly, while FBL nucleolar cap formation has previously been reported following treatment with oxaliplatin, FBL distribution has not been monitored following treatment with phenanthriplatin, to the best of our knowledge. As expected, however, treatment with phenanthriplatin at clinically relevant concentrations resulted in nucleolar cap formation, in a similar manner to ActD and oxaliplatin (Figure 5). In contrast, FBL distribution following treatment with cisplatin remained relatively unchanged (Figure 5). This highlights the potential difference in MOA of cisplatin and the nucleolar stress-inducing Pt(II) drugs. In a similar manner to phenanthriplatin, pronounced FBL nucleolar caps were observed following treatment of A549 cells with **1**–**3** for 24 h (Figure 5).

Taken together, results from the NPM1 and FBL immunofluorescence assays indicate that treatment with complexes **1**–**3** results in nucleolar protein redistribution and thus nucleolar stress responses to a similar degree as phenanthriplatin. Additionally, these complexes appear equally effective at inducing nucleolar stress, regardless of the position of the azide substituent. This suggests that small modifications to phenanthriplatin’s aromatic nitrogen donor ligand are tolerated in all three of the positions functionalized in this study.

### Treatment with Phenanthriplatin and **1**–**3** Results in Inhibition of rRNA Synthesis

**Figure 6:**
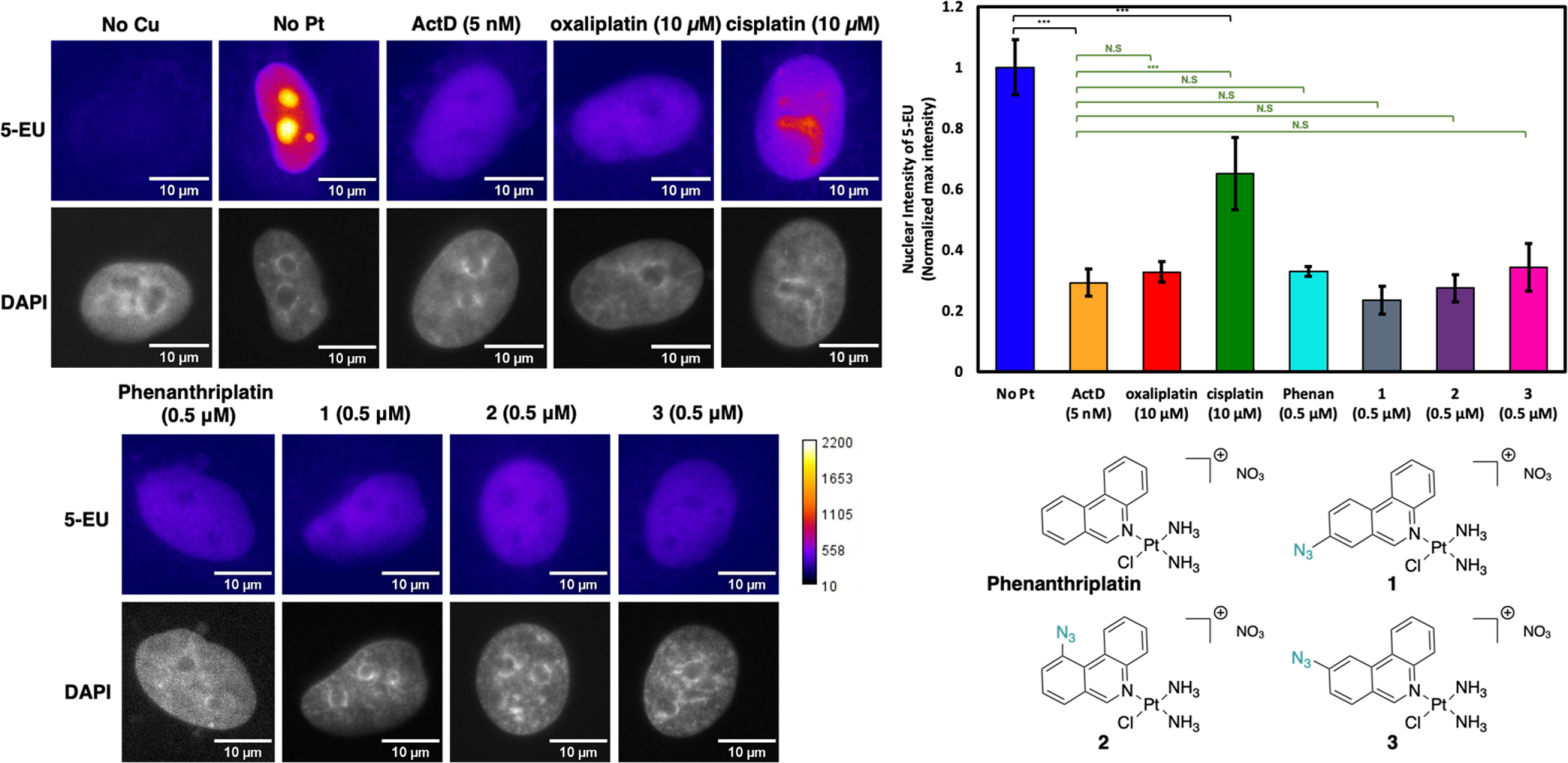
Visualization and quantification of rRNA inhibition through 5-EU (5-EU: heat map; DAPI: gray). Treatment conditions were 5 nM for ActD, 10 μM for oxaliplatin and cisplatin, and 0.5 μM for phenanthriplatin and **1**–**3** in A549 cells at 24 h treatment (*n* = 3). Scale bar = 10 μm, ***p < 0.05, N.S. *p* > 0.05.

Given the morphological changes observed in the nucleolus following treatment with phenanthriplatin and complexes **1**–**3**, we next investigated whether these changes result in impaired nucleolar function. A pulse experiment was carried out, in A549 cells treated with cisplatin, oxaliplatin, phenanthriplatin, and complexes **1**–**3** for 24 h, in which newly transcribed RNA was labeled through incorporation of 5-ethynyl uridine (5-EU) during transcription and subsequently reacted via a CuAAC reaction with an azide-containing biotin followed by binding to a fluorescent streptavidin reporter (Figure 6). Using this method, intense labeling in nucleoli, presumably of nascent rRNA, as well as diffuse labeling in the nucleoplasm, indicative of global RNA synthesis or processed rRNA, was observed. In the case of cisplatin, ∼30% inhibition of rRNA synthesis was observed at 24 h. This result is in agreement with previous ^32^P-metabolic labeling results in A549 cells that indicated some inhibition of rRNA synthesis at a 3 h time point following treatment with cisplatin, a downstream effect of the cisplatin-induced DNA damage response.^22^ In contrast to cisplatin, treatment with the recognized nucleolar stress inducers oxaliplatin and ActD resulted in a more significant reduction in rRNA transcription levels (*p* < 0.05) as observed by selective loss of EU-derived intensity in the nucleolus, a result consistent with previous reports.^22,26^

Similarly to the other known nucleolar stress inducers, treatment with phenanthriplatin resulted in a greater reduction in rRNA transcription than cisplatin (*p* < 0.05), with a similar degree of inhibition of rRNA synthesis after 24 h when compared to oxaliplatin and ActD (*p* > 0.05). While phenanthriplatin treatment has previously been shown to result in inhibition of RNA transcription, to our knowledge, this is the first example using 5-EU RNA labeling to study the drug’s effect on RNA transcription.^6^ Treatment with each of click complexes **1**–**3** was also shown to result in comparable levels of rRNA transcription inhibition to phenanthriplatin and the other nucleolar stress inducers (*p* > 0.05). As the levels of rRNA transcription observed following treatment with complexes **1**–**3** are similar to that observed following phenanthriplatin administration, this suggests that the azide modification present in complexes **1**–**3** does not prevent successful inhibition of rRNA transcription processes. Importantly, while rRNA synthesis was impaired for phenanthriplatin and complexes **1**–**3**, it is worth noting that global RNA production appeared to be still occurring following treatment, as observed by some EU-derived intensity across the nucleus.

### γH2AX Levels Following Treatment with **1**–**3** Are Similar to Phenanthriplatin

Nucleolar stress can occur as an independent stress response or as a downstream effect of DDR.^40^ H2AX phosphorylation plays a crucial role in recruiting DNA damage repair proteins and is commonly used as an indirect marker of DDR.^18^ As such, to examine whether the redistribution of NPM1 and FBL observed following treatment with **1**–**3** is due to an independent nucleolar stress response, the levels of γH2AX were monitored following treatment with **1**–**3**.

**Figure 7:**
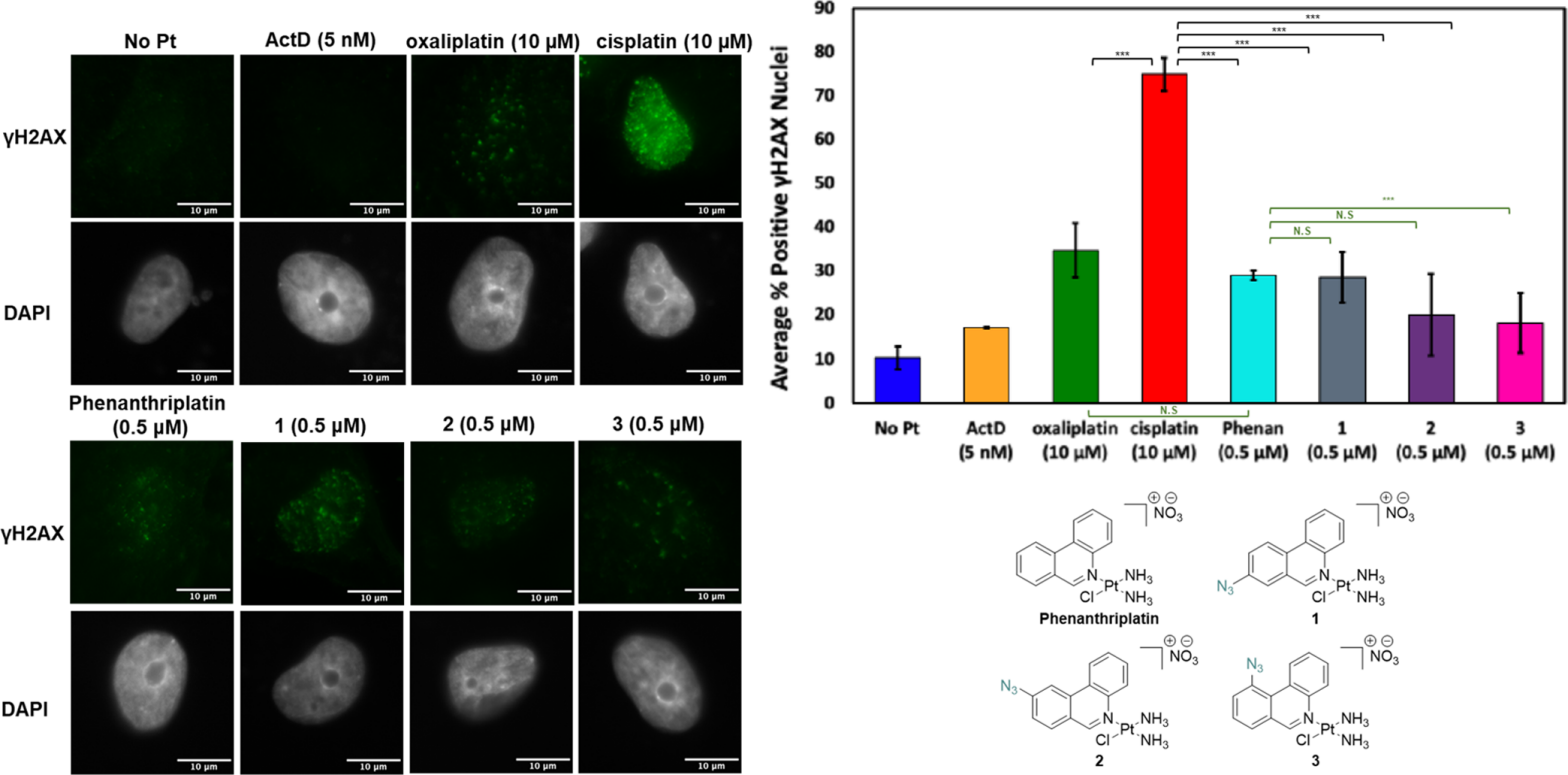
Visualization and quantification of γH2AX (γH2AX: green, DAPI: gray) as an indicator of DDR induced by **1**–**3** (0.5 μM), phenanthriplatin (0.5 μM), oxaliplatin (10 μM), cisplatin (10 μM), and ActD (5 nM) at 24 h treatments in A549 cells (*n* = 3). Standard quantification procedure described in the Experimental Section. Scale bar = 10 μm. ***p < 0.05, N.S. *p* > 0.05.

In these experiments, γH2AX levels were monitored 24 h after treatment by quantifying immunofluorescence intensities, with results reported as % of nuclei positive for γH2AX (see the Experimental Section). In line with previous results, treatment of A549 cells with the known DDR inducer, cisplatin, resulted in a strong γH2AX response indicating activation of DNA damage repair pathways (Figure 7).^17,22^

Compared to cisplatin, treatment with known nucleolar stress inducers ActD and oxaliplatin both resulted in lower levels of *y*H2AX (*p* < 0.05), in agreement with previous studies (Figure 7).^17,22^ Furthermore, *y*H2AX levels following treatment with phenanthriplatin were also significantly lower than observed following treatment with cisplatin (*p* < 0.05) (Figure 7). These results further emphasize the likely differences in mechanism of action following treatment with the different classes of Pt agents.

Similarly, to the known inducers of nucleolar stress, treatment with **1**–**3** was also found to result in significantly lower levels of *y*H2AX than cisplatin treatment (*p* < 0.05) (Figure 7). Moreover, *y*H2AX levels following treatment with **1**, **2**, or **3** were found to be similar to or lower than those following phenanthriplatin administration. Together these results indicate that treatment with **1**, **2**, and **3** causes similar *y*H2AX levels to phenanthriplatin and a less pronounced DDR than cisplatin. This result supports an independent nucleolar stress response following treatment with compounds **1**–**3**. It is worth mentioning, however, that in the case of oxaliplatin, inhibition of two DDR signaling kinases, ATM and ATR, has recently been reported to result in partial protection from nucleolar stress responses.^26^ This partial protection is observed despite low levels of H2AX phosphorylation following treatment with oxaliplatin.

This indicates that further studies with phenanthriplatin, and indeed **1**–**3**, are likely needed to fully elucidate the role of DDR in the nucleolar response of phenanthriplatin.^17,22^

### **1**–**3** Successfully Bind Hairpin DNA (HP) *In Vitro* and Can Be Functionalized by Strain-Promoted Azide–Alkyne Click Chemistry

It has previously been reported that intercalation of the phenanthridine ring of phenanthriplatin with DNA plays an important role in the formation of Pt-DNA adducts.^8−11^ As such, modifications to the phenanthridine ring of phenanthriplatin may result in impaired DNA intercalation and in turn impede Pt-DNA adduct formation. Additionally, intercalation of the azide-modified complexes **1**–**3** could result in steric hindrance that prevents efficient click-functionalization following biomolecular interactions.

**Figure 8:**
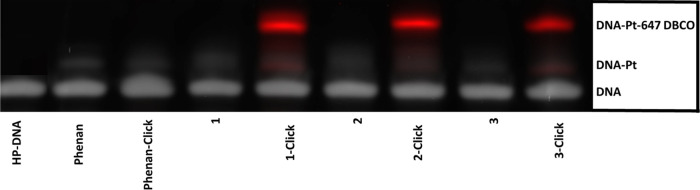
Analysis of Pt(II) complexes incubated with HP-DNA was performed by dPAGE. **1**-click, **2**-click, and **3**-click represent complexes that were first incubated with HP for 24 h followed by click reaction with Alexa-647 DBCO for an additional 24 h. Control complexes were incubated for 48 h with HP without click reaction. All samples were purified by spin column prior to dPAGE analysis. Gray: DNA stained with SYBR gold (539 nm emission wavelength), red: DNA-Pt-647 DBCO complex (671 emission wavelength).

To test the DNA-binding capability of **1**, **2**, and **3**, the complexes were incubated alongside phenanthriplatin with a HP that contains a single GG site. Successful Pt-DNA adduct formation was observed following incubation of each of **1**, **2**, **3**, and phenanthriplatin as visualized by dPAGE (Figure 8). This indicates that the small azide modification present in **1**–**3** does not prevent the complexes from binding to DNA *in vitro*. Following initial incubation of the complexes with HP-DNA, a strain-promoted azide–alkyne click reaction was carried out with the click-capable fluorophore, Alexa-647 DBCO.^17,41^ Subsequent analysis by fluorescence imaging showed successful strain-promoted azide–alkyne click reaction in the case of each of **1**, **2**, and **3**, indicating the complexes can be successfully modified following reaction with biomolecules (Figure 8). Taken together, these results indicate that **1**, **2**, and **3** are suitable click-mimics of phenanthriplatin and are suitable derivatives for studying Pt-modified biomolecules.

### *In Vitro* Cytotoxicity of **3** Is Similar to Phenanthriplatin across a Range of Cancer Cell Lines

Given similar morphological changes to the nucleolus as well as similar levels of RNA transcription inhibition following treatment with **1**–**3**, **3** was chosen as a representative example for a further *in vitro* cytotoxicity study. The *in vitro* cytotoxicities of **3**, phenanthriplatin, oxaliplatin, and cisplatin were investigated against a range of cancer cell lines after 6 days treatment (Table 1 and Figure S2).

**Table 1:** IC_50_ Values for **3**, Phenanthriplatin, Oxaliplatin, and Cisplatin at 6 days, as Determined by Acid Phosphatase Assay

IC50 (μM)	**3**	phenanthriplatin	oxaliplatin	cisplatin
A549	0.203 ± 0.023	0.201 ± 0.022	1.725 ± 0.434	2.096 ± 0.441
PANC-1	0.092 ± 0.021	0.096 ± 0.025	4.389 ± 0.943	3.794 ± 0.837
SK-LU-1	0.211 ± 0.022	0.327 ± 0.032	2.806 ± 0.582	0.659 ± 0.198

In line with the results obtained for nucleolar protein redistribution, RNA transcription inhibition, and γH2AX levels, **3** was found to exhibit similar *in vitro* cytotoxic activity to phenanthriplatin in the A549 (nonsmall cell lung) cell line. Furthermore, **3** was found to have similar activity to phenanthriplatin in the PANC-1 (pancreatic) cell line tested, while also showing greater activity in the SK-LU-1 (lung) cell line. Previous studies have shown that phenanthriplatin is highly effective against a range of lung and pancreatic cancer cell lines, and as such, these results are in close agreement with those previously reported.^6,28^ These results indicate that early cellular processing of **3** and the mechanisms of action activated by the complex ultimately lead to similar *in vitro* cytotoxic activity as the parent complex in the cell lines tested.

## Conclusions

We have reported the design, synthesis, and characterization of **1**–**3**, the first azide-containing phenanthriplatin derivatives reported to date. Through monitoring NPM1 localization, FBL cap formation, and RNA transcription levels, we have shown that each of the three complexes induce redistribution of nucleolar proteins and disruption to nucleolar morphology to a similar degree as phenanthriplatin, while also retaining the ability to inhibit RNA synthesis. Furthermore, by measuring *y*H2AX phosphorylation levels following treatment with **1**–**3**, we have shown that these complexes induce a level of DDR activation similar to that of phenanthriplatin. Through *in vitro* DNA hairpin incubation, we have shown that **1**–**3** can successfully form adducts with DNA and can subsequently be functionalized with a fluorescent reporter through strain-promoted azide–alkyne click chemistry. Finally, the cytotoxicity of **3** was shown to be similar to phenanthriplatin in a range of cancer cell lines, emphasizing the similarity between the reported click-capable phenanthriplatin mimic and the parent complex, phenanthriplatin. As such, we present **1**–**3** as suitable click-capable phenanthriplatin mimics for future studies focused on Pt-induced nucleolar stress and better understanding of Pt-based cell death pathways.

### Cell Culture and Treatment

A549 human lung carcinoma cells (#CCL-185, American Type Culture Collection) were cultured in 5% CO_2_ at 37 °C in Dulbecco’s modified Eagle’s medium (DMEM) with 10% fetal bovine serum (FBS) and 1% antibiotic-antimycotic. Treatments were performed on cells that had been grown for 11–26 passages to 70% confluency. All treatments were performed for 24 h at 10 μM for oxaliplatin, 0.5 μM concentrations for phenanthriplatin, **1**, **2**, and **3**. Oxaliplatin was made in a 5 mM stock solution, while phenanthriplatin, **1**, **2**, and **3** were made into a 5 mM stock solution and further diluted to a 250 μM stock solution. The complex stock solutions were made with dimethylformamide (DMF) (phenanthriplatin, **1**, **2**, and **3**), water (oxaliplatin), or DMSO (ActD). Stock solutions were diluted into media immediately prior to drug treatment. Treatments were performed in triplicate, and additional replicates are available from the corresponding author upon reasonable request.

### Immunofluorescence

Cells were grown on coverslips (Ted Pella product no. 260368, round glass coverslips, 10 mm diameter 0.16–0.19 mm thick) as described above. After treatment was complete, cells were washed with phosphate-buffered saline (PBS) and fixed with 4% paraformaldehyde (PFA) in PBS for 20 min at room temperature (RT). PFA was removed using aspiration, and cells were permeabilized with 0.5% Triton-X in PBS for 20 min at RT. Two 10 min blocking steps were then performed with 1% bovine serum albumin (BSA) in poly(butylenes succinate-*co*-terephthalate) (PBST) (PBS with 0.1% Tween-20). The cells were incubated for 1 h in primary antibody NPM1 or γH2AX for DDR and for an hour and a half with the primary antibody Fibrillarin for nucleolar stress response (NPM1 monoclonal antibody, FC-61991, Thermo Fisher, 1:800 dilution in PBST with 1% BSA) (Phospho-Histone H2A.X, Ser139) Monoclonal Antibody (CR55T33, Thermo Fisher, 2.5 μg in PBST with 1% BSA), (anti-Fibrillarin antibody ab4566 from Abcam, 1:400 dilution) and 1 h in secondary antibody for NPM1 or γH2AX and 1.5 h for fibrillarin (Goat Anti-Mouse IGG H&L Alexa Fluor 488, ab150113, Abcam, 1:1000 dilution in PBST with 1% BSA), with three 5 min wash steps using PBST between antibody incubations. It was washed again in the same manner before mounting the slides. Coverslips were then mounted on slides with ProLong Diamond Antifade Mountant with DAPI (Thermo Fisher) according to the manufacturer’s instructions.

### Labeling of RNA with 5-Ethynyl Uridine (5-EU)

Cells were grown on coverslips (Ted Pella product no. 260368, round glass coverslips, 10 mm diameter 0.16–0.19 mm thick) as described above. The cells were treated with the compound of interest for a total of 24 h. For RNA labeling, the cells were washed with PBS 3× at the 20 h mark, and media containing both the compound and 2 mM EU were added to the cells and incubated for 4 h. Following treatment, the cells were washed with PBS 3× for 5 min each and fixed with 4% PFA in PBS for 20 min. The cells were then permeabilized with 0.5% Triton-X in PBS for 20 min. Block was performed with 5% BSA in PBST for 1 h. During the last 10 min of blocking, a click cocktail containing 10 mM sodium ascorbate, 100 μM azide-PEG3-biotin (Click Chemistry Tools, AZ104-5), and premixed 2 mM CuSO_4_ and 4 mM THPTA in PBS was made and then added to cells for 1 h. Control treatments for no Cu had all components of the click cocktail added, excluding the CuSO_4_. After the click reaction, the cells were washed 5× with PBST for 5 min each. The coverslips were then incubated with 5 μg/mL streptavidin, Alexa Fluor 488 conjugate (Invitrogen, S11223) in 5% BSA in PBST for 1 h. The cells were then washed with 0.5% Triton-X in PBS 1× and in PBS 2× for 5 min each. The coverslips were mounted on slides with antifade fluorescence mounting media (Abcam) and left to cure overnight before imaging. All of the above steps were performed at RT.

### Image Processing and Quantification

The quantification of NPM1 relocalization was performed in an automated fashion by using a Python 3 script. Images were preprocessed in ImageJ,^42,43^ to convert the DAPI and NPM1 channels into separate 16-bit grayscale images. Between 50 and 250 cells were analyzed for each treatment group. Nuclei were segmented using the DAPI images using Li thresholding function in the Scikit-Image Python package.^44^ The coefficient of variation (CV) for individual nuclei, which is defined as the standard deviation in pixel intensity divided by the mean pixel intensity, was calculated from the NPM1 images by using the SciPy Python package. All of the data was normalized to the no-treatment in each experiment. NPM1 imaging results for each complex were observed in triplicate. Data are represented as boxplots generated using Seaborn within Python.

Quantification of γH2AX intensity and foci was performed with CellProfiler 4.2.1 software.^45^ In one analysis method, a “percent positive” value was calculated for each treatment condition relative to the untreated control. A threshold was determined for a positive γH2AX result based on the 90th percentile intensity value of the untreated control for each time point. Nuclei in the experimental samples with integrated intensity levels higher than this were counted as positive for γH2AX. Significance testing was done via a *t*-test to obtain a *p*-value.

### *In Vitro* DNA Gel Binding and Fluorophore Clicking

Hairpin DNA sequence (TATGGTATTTTTATACCATA) (280 μM) was folded by rapid heating to 90 °C and slow cooling to 4 °C in 10 mM Na_2_HPO_4_/NaH_2_PO_4_ buffer (pH 7.1), 0.1 M NaNO_3_, and 10 mM Mg(NO_3_)_2_. The platinum complex (830 μM) was then added, and the solution was incubated at 37 °C for 24 h. For click complexes, 195 μM Alexa-657 DBCO (AZDye) was added and incubated at 37 °C for an additional 24 h. Nonclick control complexes were incubated for an additional 24 h. at 37 °C. All complexes were then purified with Sephadex G-25 Medium size exclusion resin (GE Healthcare) on laboratory-prepared spin columns (BioRad) to remove unbound platinum and fluorophore. Purified samples were added at a DNA concentration of 200 ng on dPAGE (19:1 20% acrylamide in 8 M urea) and ran at 180 V for 2 h. Gels were then stained with SYBR gold for 5 min and imaged using a GE Amersham Typhoon gel imager.

### *In Vitro* Proliferation Assay

A549, SK-LU-1 (lung adenocarcinoma), and Caco-2 (colorectal adenocarcinoma) cancer cell lines were maintained in RPMI 1640 medium supplemented with 10% fetal bovine serum (PAA Laboratories, Austria). PANC-1 cells (pancreatic carcinoma) were maintained in Dulbecco’s modified Eagle’s medium (DMEM) supplemented with 5% fetal bovine serum (Gibco) and 2% l-glutamine (Sigma, St Louis, MO). All cell lines were kept at 37 °C in a 5% CO_2_, 95% air-humidified incubator. The cells were cultured in 96-well flat-bottom plates for 24 h before they were exposed to a range of concentrations of the targeted therapies for 6 days. The cell densities varied from 0.6 × 10^4^ cells/mL (Caco-2) to 1 × 10^4^ cells/mL (A549) and 2 × 10^4^ cells/mL (SK-LU-1 and PANC-1). The percentage cell survival was then determined using an acid phosphatase assay. Briefly, media was removed from the plates and each well was washed twice with PBS. The cells were exposed to 10 mM PNP substrate in 0.1 M sodium acetate for approximately 1 h. The reaction was stopped using 1 M NaOH. and the plates were read at 405 and 620 nm on a plate reader. The percentage cell survival was calculated as a percentage relative to a nontreated control.

### Synthesis

Oxaliplatin and cisplatin were purchased from TCI. Unless otherwise noted, starting materials were purchased from Millipore Sigma-Aldrich, TCI, or BLD Pharm. Phenanthriplatin was synthesized according to previously published methods.^6^

### General Synthesis of **1**–**3**

AgNO_3_ (0.17 g, 1.00 mmol) was added to a solution of cisplatin (0.30 g, 1.00 mmol) in 15 mL of DMF. The reaction mixture was then stirred in the dark at 55 °C overnight. Following stirring, precipitated AgCl was removed by filtration through Celite. Azidophenanthridine (0.20 g, 0.90 mmol) was then added to the filtrate and the reaction mixture was stirred for a further 24 h at 55 °C. On returning, the solvent was removed under reduced pressure and the residue resuspended in approximately 30 mL of methanol. The mixture was then filtered, and the crude product precipitated by adding 100 mL of diethyl ether to the filtrate. The crude product was further purified by redissolving in methanol and recrystallization from a methanol diethyl ether solution. The final compound was isolated by vacuum filtration, washed with diethyl ether, and dried under reduced pressure.
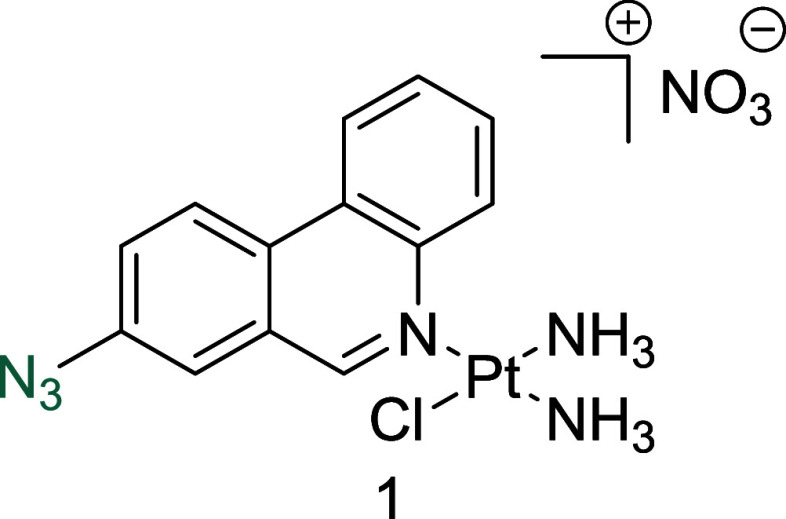


#### *Cis*-[Pt(NH_3_)_2_(8-Azidophenanthridine)Cl]NO_3_, **1**

Yellow crystals were obtained following recrystallization from MeOH/Et_2_O (88 mg, 18%). ^1^H NMR (400 MHz, DMSO-*d*_6_) δ 9.94 (s, 1H), 9.75 (d, 1H, *J* = 8.3 Hz), 8.99 (d, 1H, *J* = 9.0 Hz), 8.89 (d, 1H, *J* = 8.1 Hz), 8.25 (s, 1H), 8.01 (t, 1H, *J* = 7.5 Hz), 7.90 (m, 2H), 4.56 (s, 3H), 4.44 (s, 3H). ^13^C NMR (101 MHz, MeOD-*d*_4_) δ: 160.5, 143.7, 142.4, 130.8, 130.5, 130.4, 130.3, 128.9, 127.2, 126.9, 125.7, 124.0, 118.9. HRMS (ESI^+^) (MeOH) [M – NO_3_]^+^: *m*/*z* calcd for C_13_H_14_ClN_6_Pt: 484.0611, found: 484.0624.
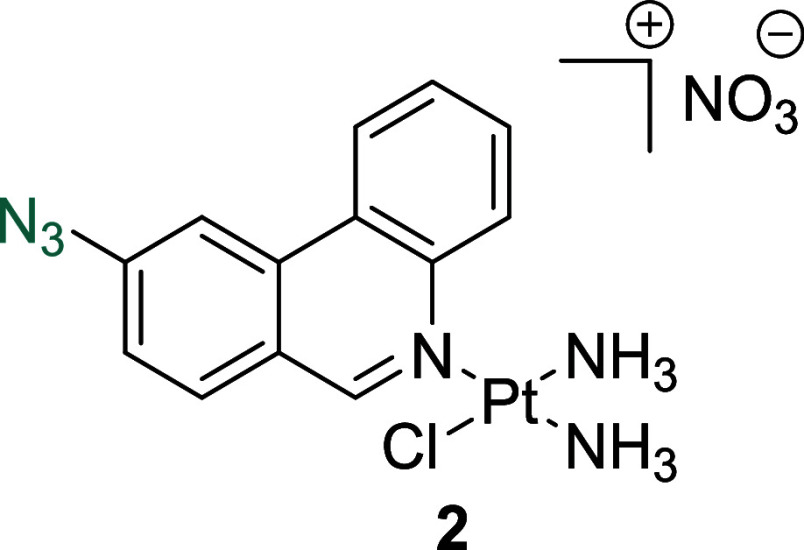


#### *Cis*-[Pt(NH_3_)_2_(9-Azidophenanthridine)Cl]NO_3_, **2**

An off-white solid was obtained following recrystallization from MeOH/Et_2_O (71 mg, 14%). ^1^H NMR (400 MHz, DMSO-*d*_6_) δ 9.87 (s, 1H), 9.75 (d, 1H, *J* = 8.4 Hz), 8.96 (d, 1H, *J* = 8.2 Hz), 8.57 (s, 1H), 8.48 (d, 1H, *J* = 8.6 Hz), 8.02 (t, 1H, *J* = 7.6 Hz), 7.88 (t, 1H, *J* = 7.5 Hz), 7.67 (d, 1H, *J* = 8.4 Hz), 4.63 (s, 3H), 4.48 (s, 3H). ^13^C NMR (101 MHz, DMSO-*d*_6_) δ: 159.1, 145.7, 142.6, 133.4, 132.3, 129.8, 129.2, 128.5, 124.5, 123.8, 123.5, 121.2, 111.7. HRMS (ESI^+^) (MeOH) [M – NO_3_]^+^: *m*/*z* calcd for C_13_H_14_ClN_6_Pt: 484.0611, found: 484.0627.
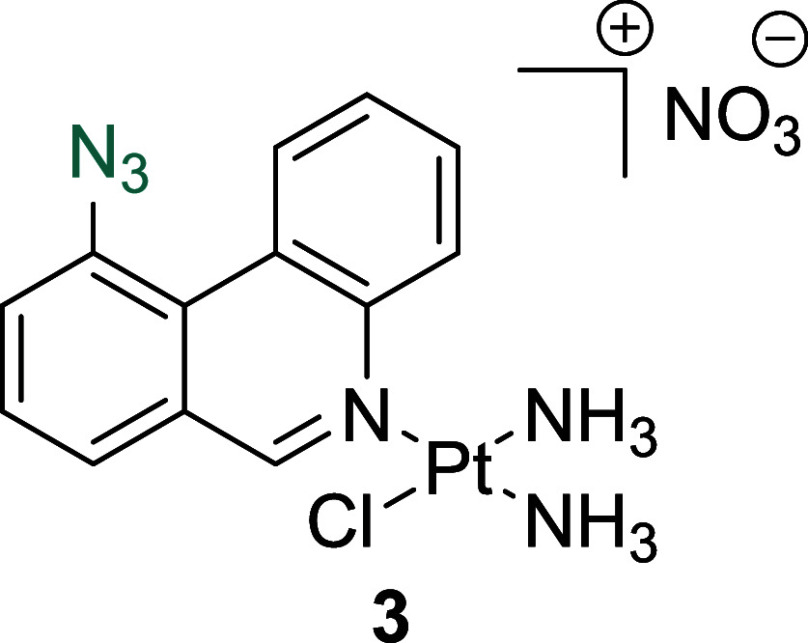


#### *Cis*-[Pt(NH_3_)_2_(10-Azidophenanthridine)Cl]NO_3_, **3**

A white solid was obtained following recrystallization from MeOH/Et_2_O (122 mg, 25%). ^1^H NMR (400 MHz, DMSO-*d*_6_) δ 9.95 (s, 1H), 9.89 (dd, 1H, *J* = 8.4, 0.8 Hz), 9.76 (dd, 1H *J* = 8.5, 0.8 Hz), 8.31 (d, 1H, *J* = 7.1 Hz), 8.12 (dd, 1H, *J* = 7.8, 0.9 Hz), 8.02 (m, 2H), 7.94–7.88 (m, 1H), 4.57 (s, 3H), 4.46 (s, 3H). ^13^C NMR (101 MHz, DMSO-*d*_6_) δ: 153.6, 144.7, 136.3, 129.8, 128.7, 128.3, 128.1, 127.0, 127.0, 126.1, 122.8, 122.5, 122.4. HRMS (ESI^+^) (MeOH) [M – NO_3_]^+^: *m*/*z* calcd for C_13_H_14_ClN_6_Pt: 484.0611, found: 484.0627.

## Supporting Information Available

Materials and methods, including additional synthetic methods, characterization data for complexes **1**–**3**, NPM1 relocalization cell images, and single-crystal X-ray diffraction data (PDF)

## References

[ref1] Forgie B. N.; Prakash R.; Telleria C. M. Revisiting the Anti-Cancer Toxicity of Clinically Approved Platinating Derivatives. Int. J. Mol. Sci. 2022, 23, 15410 10.3390/ijms232315410. 36499737 PMC9793759

[ref2] Rottenberg S.; Disler C.; Perego P. The rediscovery of platinum-based cancer therapy. Nat. Rev. Cancer 2021, 21 (1), 37–50. 10.1038/s41568-020-00308-y. 33128031

[ref3] O’Dowd P. D.; Sutcliffe D. F.; Griffith D. M. Oxaliplatin and its derivatives—An overview. Coord. Chem. Rev. 2023, 497, 215439 10.1016/j.ccr.2023.215439.

[ref4] Fanelli M.; Formica M.; Fusi V.; Giorgi L.; Micheloni M.; Paoli P. New trends in platinum and palladium complexes as antineoplastic agents. Coord. Chem. Rev. 2016, 310, 41–79. 10.1016/j.ccr.2015.11.004.

[ref5] Brabec V.; Kašpárková J.; Vrána O.; Nováková O.; Cox J. W.; Qu Y.; Farrell N. DNA Modifications by a Novel Bifunctional Trinuclear Platinum Phase I Anticancer Agent. Biochemistry 1999, 38 (21), 6781–6790. 10.1021/bi990124s. 10346899

[ref6] Park G. Y.; Wilson J. J.; Song Y.; Lippard S. J. Phenanthriplatin, a monofunctional DNA-binding platinum anticancer drug candidate with unusual potency and cellular activity profile. Proc. Natl. Acad. Sci. U.S.A. 2012, 109 (30), 11987–92. 10.1073/pnas.1207670109. 22773807 PMC3409760

[ref7] Zhou W.; Almeqdadi M.; Xifaras M. E.; Riddell I. A.; Yilmaz Ö. H.; Lippard S. J. The effect of geometric isomerism on the anticancer activity of the monofunctional platinum complex trans-[Pt(NH(3))(2)(phenanthridine)Cl]NO(3). Chem. Commun. 2018, 54 (22), 2788–2791. 10.1039/C8CC00393A. PMC5988251 29484327

[ref8] Veclani D.; Melchior A.; Tolazzi M.; Cerón-Carrasco J. P. Using Theory To Reinterpret the Kinetics of Monofunctional Platinum Anticancer Drugs: Stacking Matters. J. Am. Chem. Soc. 2018, 140 (43), 14024–14027. 10.1021/jacs.8b07875. 30185041

[ref9] Almaqwashi A. A.; Zhou W.; Naufer M. N.; Riddell I. A.; Yilmaz Ö. H.; Lippard S. J.; Williams M. C. DNA Intercalation Facilitates Efficient DNA-Targeted Covalent Binding of Phenanthriplatin. J. Am. Chem. Soc. 2019, 141 (4), 1537–1545. 10.1021/jacs.8b10252. 30599508 PMC6491043

[ref10] Dabbish E.; Russo N.; Sicilia E. Rationalization of the Superior Anticancer Activity of Phenanthriplatin: An In-Depth Computational Exploration. Chem. - Eur. J. 2020, 26 (1), 259–268. 10.1002/chem.201903831. 31614021

[ref11] Veclani D.; Tolazzi M.; Cerón-Carrasco J. P.; Melchior A. Intercalation Ability of Novel Monofunctional Platinum Anticancer Drugs: A Key Step in Their Biological Action. J. Chem. Inf. Model. 2021, 61 (9), 4391–4399. 10.1021/acs.jcim.1c00430. 34156233 PMC8479807

[ref12] Kellinger M. W.; Park G. Y.; Chong J.; Lippard S. J.; Wang D. Effect of a Monofunctional Phenanthriplatin-DNA Adduct on RNA Polymerase II Transcriptional Fidelity and Translesion Synthesis. J. Am. Chem. Soc. 2013, 135 (35), 13054–13061. 10.1021/ja405475y. 23927577 PMC3791135

[ref13] Gregory M. T.; Park G. Y.; Johnstone T. C.; Lee Y. S.; Yang W.; Lippard S. J. Structural and mechanistic studies of polymerase η bypass of phenanthriplatin DNA damage. Proc. Natl. Acad. Sci. U.S.A. 2014, 111 (25), 9133–9138. 10.1073/pnas.1405739111. 24927576 PMC4078841

[ref14] Riddell I. A.; Agama K.; Park G. Y.; Pommier Y.; Lippard S. J. Phenanthriplatin Acts As a Covalent Poison of Topoisomerase II Cleavage Complexes. ACS Chem. Biol. 2016, 11 (11), 2996–3001. 10.1021/acschembio.6b00565. 27648475 PMC5248983

[ref15] Urankar D.; Košmrlj J. Preparation of diazenecarboxamide–carboplatin conjugates by click chemistry. Inorg. Chim. Acta 2010, 363 (14), 3817–3822. 10.1016/j.ica.2010.07.031.

[ref16] Wirth R.; White J. D.; Moghaddam A. D.; Ginzburg A. L.; Zakharov L. N.; Haley M. M.; DeRose V. J. Azide vs Alkyne Functionalization in Pt(II) Complexes for Post-treatment Click Modification: Solid-State Structure, Fluorescent Labeling, and Cellular Fate. J. Am. Chem. Soc. 2015, 137 (48), 15169–15175. 10.1021/jacs.5b09108. 26512733

[ref17] Guerrero A. S.; O’Dowd P. D.; Pigg H. C.; Alley K. R.; Griffith D. M.; DeRose V. J. Comparison of click-capable oxaliplatin and cisplatin derivatives to better understand Pt(ii)-induced nucleolar stress. RSC Chem. Biol. 2023, 4, 785–793. 10.1039/D3CB00055A. 37799581 PMC10549245

[ref18] Bruno P. M.; Liu Y.; Park G. Y.; Murai J.; Koch C. E.; Eisen T. J.; Pritchard J. R.; Pommier Y.; Lippard S. J.; Hemann M. T. A subset of platinum-containing chemotherapeutic agents kills cells by inducing ribosome biogenesis stress. Nat. Med. 2017, 23 (4), 461–471. 10.1038/nm.4291. 28263311 PMC5520548

[ref19] Yang K.; Yang J.; Yi J. Nucleolar Stress: hallmarks, sensing mechanism and diseases. Cell Stress 2018, 2 (6), 125–140. 10.15698/cst2018.06.139. 31225478 PMC6551681

[ref20] Ozdian T.; Holub D.; Maceckova Z.; Varanasi L.; Rylova G.; Rehulka J.; Vaclavkova J.; Slavik H.; Moudry P.; Znojek P.; Stankova J.; de Sanctis J. B.; Hajduch M.; Dzubak P. Proteomic profiling reveals DNA damage, nucleolar and ribosomal stress are the main responses to oxaliplatin treatment in cancer cells. J. Proteomics 2017, 162, 73–85. 10.1016/j.jprot.2017.05.005. 28478306

[ref21] Sutton E. C.; McDevitt C. E.; Prochnau J. Y.; Yglesias M. V.; Mroz A. M.; Yang M. C.; Cunningham R. M.; Hendon C. H.; DeRose V. J. Nucleolar Stress Induction by Oxaliplatin and Derivatives. J. Am. Chem. Soc. 2019, 141 (46), 18411–18415. 10.1021/jacs.9b10319. 31670961 PMC7735645

[ref22] Sutton E. C.; DeRose V. J. Early nucleolar responses differentiate mechanisms of cell death induced by oxaliplatin and cisplatin. J. Biol. Chem. 2021, 296, 100633 10.1016/j.jbc.2021.100633. 33819479 PMC8131322

[ref23] McDevitt C. E.; Guerrero A. S.; Smith H. M.; DeRose V. J. Influence of Ring Modifications on Nucleolar Stress Caused by Oxaliplatin-Like Compounds. ChemBioChem 2022, 23 (14), e202200130 10.1002/cbic.202200130. 35475312

[ref24] Pigg H. C.; Yglesias M. V.; Sutton E. C.; McDevitt C. E.; Shaw M.; DeRose V. J. Time-Dependent Studies of Oxaliplatin and Other Nucleolar Stress-Inducing Pt(II) Derivatives. ACS Chem. Biol. 2022, 17 (8), 2262–2271. 10.1021/acschembio.2c00399. 35917257

[ref25] Schmidt H. B.; Jaafar Z. A.; Wulff B. E.; Rodencal J. J.; Hong K.; Aziz-Zanjani M. O.; Jackson P. K.; Leonetti M. D.; Dixon S. J.; Rohatgi R.; Brandman O. Oxaliplatin disrupts nucleolar function through biophysical disintegration. Cell Rep. 2022, 41 (6), 111629 10.1016/j.celrep.2022.111629. 36351392 PMC9749789

[ref26] Nechay M.; Wang D.; Kleiner R. E. Inhibition of nucleolar transcription by oxaliplatin involves ATM/ATR kinase signaling. Cell Chem. Biol. 2023, 30 (8), 906–919.e4. 10.1016/j.chembiol.2023.06.010. 37433295 PMC10529435

[ref27] McDevitt C. E.; Yglesias M. V.; Mroz A. M.; Sutton E. C.; Yang M. C.; Hendon C. H.; DeRose V. J. Monofunctional platinum(II) compounds and nucleolar stress: is phenanthriplatin unique?. J. Biol. Inorg. Chem. 2019, 24 (6), 899–908. 10.1007/s00775-019-01707-9. 31494760 PMC7660985

[ref28] Mitchell R. J.; Kriger S. M.; Fenton A. D.; Havrylyuk D.; Pandeya A.; Sun Y.; Smith T.; DeRouchey J. E.; Unrine J. M.; Oza V.; Blackburn J. S.; Wei Y.; Heidary D. K.; Glazer E. C. A monoadduct generating Ru(ii) complex induces ribosome biogenesis stress and is a molecular mimic of phenanthriplatin. RSC Chem. Biol. 2023, 4 (5), 344–353. 10.1039/D2CB00247G. 37181632 PMC10170627

[ref29] Farrer N. J.; Griffith D. M. Exploiting azide–alkyne click chemistry in the synthesis, tracking and targeting of platinum anticancer complexes. Curr. Opin. Chem. Biol. 2020, 55, 59–68. 10.1016/j.cbpa.2019.12.001. 31945705 PMC7254056

[ref30] Sutton E. C.; McDevitt C. E.; Yglesias M. V.; Cunningham R. M.; DeRose V. J. Tracking the cellular targets of platinum anticancer drugs: Current tools and emergent methods. Inorg. Chim. Acta 2019, 498, 118984 10.1016/j.ica.2019.118984.

[ref31] Cunningham R. M.; DeRose V. J. Platinum Binds Proteins in the Endoplasmic Reticulum of S. cerevisiae and Induces Endoplasmic Reticulum Stress. ACS Chem. Biol. 2017, 12 (11), 2737–2745. 10.1021/acschembio.7b00553. 28892625

[ref32] Osborn M. F.; White J. D.; Haley M. M.; DeRose V. J. Platinum-RNA Modifications Following Drug Treatment in S. cerevisiae Identified by Click Chemistry and Enzymatic Mapping. ACS Chem. Biol. 2014, 9 (10), 2404–2411. 10.1021/cb500395z. 25055168 PMC4201330

[ref33] Moghaddam A. D.; White J. D.; Cunningham R. M.; Loes A. N.; Haley M. M.; DeRose V. J. Convenient detection of metal–DNA, metal–RNA, and metal–protein adducts with a click-modified Pt(ii) complex. Dalton Trans. 2015, 44 (8), 3536–3539. 10.1039/C4DT02649G. 25338004

[ref34] White J. D.; Guzman L. E.; Zakharov L. N.; Haley M. M.; DeRose V. J. An Alkyne-Appended, Click-Ready PtII Complex with an Unusual Arrangement in the Solid State. Angew. Chem., Int. Ed. 2015, 54 (3), 1032–1035. 10.1002/anie.201409853. 25429919

[ref35] Moretton A.; Slyskova J.; Simaan M. E.; Arasa-Verge E. A.; Meyenberg M.; Cerrón-Infantes D. A.; Unterlass M. M.; Loizou J. I. Clickable Cisplatin Derivatives as Versatile Tools to Probe the DNA Damage Response to Chemotherapy. Front. Oncol. 2022, 12, 874201 10.3389/fonc.2022.874201. 35719993 PMC9202558

[ref36] Cunningham R. M.; Hickey A. M.; Wilson J. W.; Plakos K. J. I.; DeRose V. J. Pt-induced crosslinks promote target enrichment and protection from serum nucleases. J. Inorg. Biochem. 2018, 189, 124–133. 10.1016/j.jinorgbio.2018.09.007. 30245274 PMC7703794

[ref37] Hennessy J.; McGorman B.; Molphy Z.; Farrell N. P.; Singleton D.; Brown T.; Kellett A. A Click Chemistry Approach to Targeted DNA Crosslinking with cis-Platinum(II)-Modified Triplex-Forming Oligonucleotides. Angew. Chem., Int. Ed. 2022, 61 (3), e202110455 10.1002/anie.202110455. PMC9299770 34652881

[ref38] Johnstone T. C.; Lippard S. J. The Chiral Potential of Phenanthriplatin and Its Influence on Guanine Binding. J. Am. Chem. Soc. 2014, 136 (5), 2126–2134. 10.1021/ja4125115. 24417436 PMC3937553

[ref39] van Sluis M.; McStay B. Nucleolar reorganization in response to rDNA damage. Curr. Opin. Cell Biol. 2017, 46, 81–86. 10.1016/j.ceb.2017.03.004. 28431265

[ref40] Koike A.; Nishikawa H.; Wu W.; Okada Y.; Venkitaraman A. R.; Ohta T. Recruitment of Phosphorylated NPM1 to Sites of DNA Damage through RNF8-Dependent Ubiquitin Conjugates. Cancer Res. 2010, 70 (17), 6746–6756. 10.1158/0008-5472.CAN-10-0382. 20713529

[ref41] Eeftens J. M.; van der Torre J.; Burnham D. R.; Dekker C. Copper-free click chemistry for attachment of biomolecules in magnetic tweezers. BMC Biophys. 2015, 8 (1), 9 10.1186/s13628-015-0023-9. 26413268 PMC4582843

[ref42] Schindelin J.; Arganda-Carreras I.; Frise E.; Kaynig V.; Longair M.; Pietzsch T.; Preibisch S.; Rueden C.; Saalfeld S.; Schmid B.; Tinevez J.-Y.; White D. J.; Hartenstein V.; Eliceiri K.; Tomancak P.; Cardona A. Fiji: an open-source platform for biological-image analysis. Nat. Methods 2012, 9 (7), 676–682. 10.1038/nmeth.2019. 22743772 PMC3855844

[ref43] Rueden C. T.; Schindelin J.; Hiner M. C.; DeZonia B. E.; Walter A. E.; Arena E. T.; Eliceiri K. W. ImageJ2: ImageJ for the next generation of scientific image data. BMC Bioinf. 2017, 18 (1), 529 10.1186/s12859-017-1934-z. PMC5708080 29187165

[ref44] van der Walt S.; Schönberger J. L.; Nunez-Iglesias J.; Boulogne F.; Warner J. D.; Yager N.; Gouillart E.; Yu T. scikit-image: image processing in Python. PeerJ 2014, 2, e453 10.7717/peerj.453. 25024921 PMC4081273

[ref45] Carpenter A. E.; Jones T. R.; Lamprecht M. R.; Clarke C.; Kang I. H.; Friman O.; Guertin D. A.; Chang J. H.; Lindquist R. A.; Moffat J.; Golland P.; Sabatini D. M. CellProfiler: image analysis software for identifying and quantifying cell phenotypes. Genome Biol. 2006, 7 (10), R100 10.1186/gb-2006-7-10-r100. 17076895 PMC1794559

