# Supplementary material for: Click-Capable Phenanthriplatin Derivatives as Tools to Study Pt(II)-Induced Nucleolar Stress
Source: ACS Chem Biol. 2024 Mar 14;19(4):875–85. doi: 10.1021/acschembio.3c00607 (PMC11040607; doi:10.1021/acschembio.3c00607)
Supplement: Supplementary file 1 — cb3c00607_si_001.pdf [file cb3c00607_si_001.pdf]

Supporting Information

Click-Capable Phenanthriplatin Derivatives as Tools to Study Pt(II)-Induced Nucleolar Stress

Paul D. O'Dowd,[a,b] Andres S. Guerrero,[c] Katelyn R. Alley,[c] Hannah C. Pigg,[c] Fiona O'Neill,[d] Justine Meiller,[d] Chloe Hobbs,[a] Daniel A. Rodrigues,[a] Brendan Twamley,[e] Finbarr O'Sullivan,[d] Victoria J. DeRose,[c] Darren M. Griffith*[a,b].

[a] Department of Chemistry, Royal College of Surgeons in Ireland, Dublin, Ireland

[b] SSPC, the Science Foundation Ireland Research Centre for Pharmaceuticals

[c] Department of Chemistry and Biochemistry, University of Oregon, Eugene, OR, USA

[d] Life Science Institute, Dublin City University, Dublin, Ireland.

[e] Department of Chemistry, Trinity College Dublin, Dublin, Ireland.

Corresponding Author Email Address: dgriffith@rcsi.ie

Contents

NPM1 Average Coefficient of Variation for 1,2 and 3: 2

In-vitro activity of 3 and Pt controls: 3

Synthesis & Characterization of Ligands: 4

SI NMR (^{1}H & ^{13}C) and HRMS Spectra for 1, 2 and 3: 8

Single crystal X-Ray diffraction: 14

NPM1 Average Coefficient of Variation for 1,2 and 3:

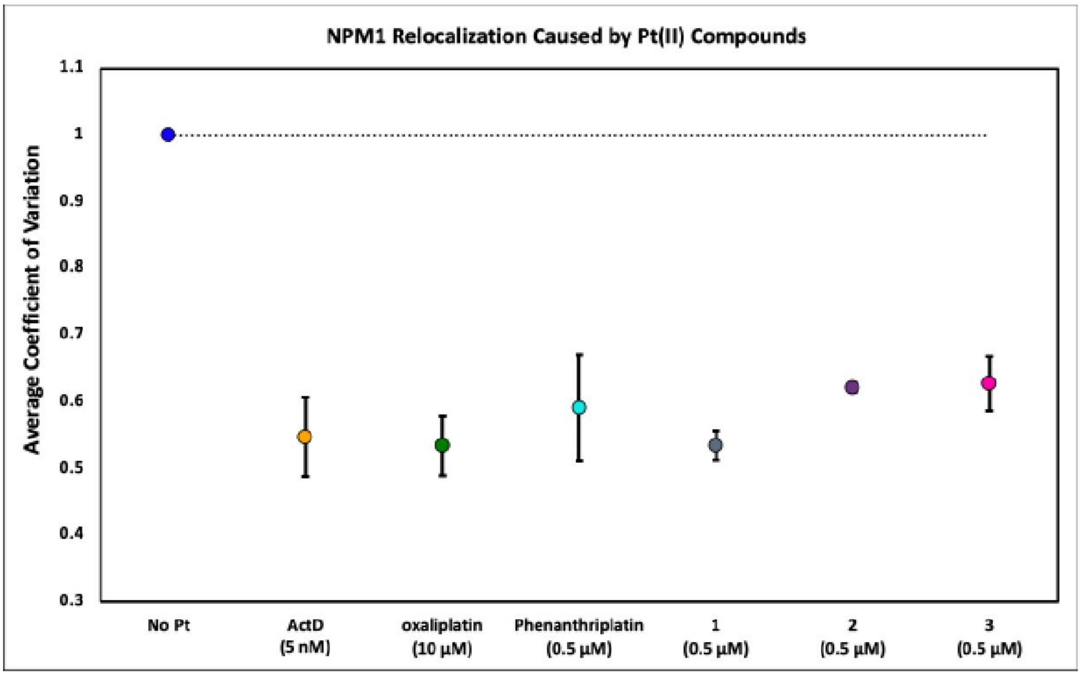

A549 cells, 24 hr treatment (**oxaliplatin**: 10 μM, **Phenanthriplatin**, **1**, **2**, and **3**: 0.5 μM, **ActD**: 5 nM), average of 3 trials and std.

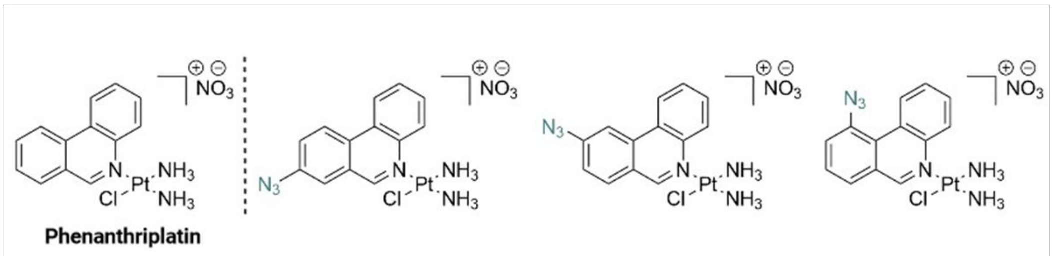

Figure S1. Average Coefficient of Variation (CV) quantification of NPM1 relocalization induced by **1**, **2** and **3**.Treatment conditions indicated (either 10 μM or 0.5 μM for Pt complexes, 5 nM for ActD) in A549 cells at 24 hr treatment; CV calculations, and boxplot presentations as described in the Experimental Section. For each treatment data set, average of CV of 3 trials along with std.

In-vitro activity of 3 and Pt controls:

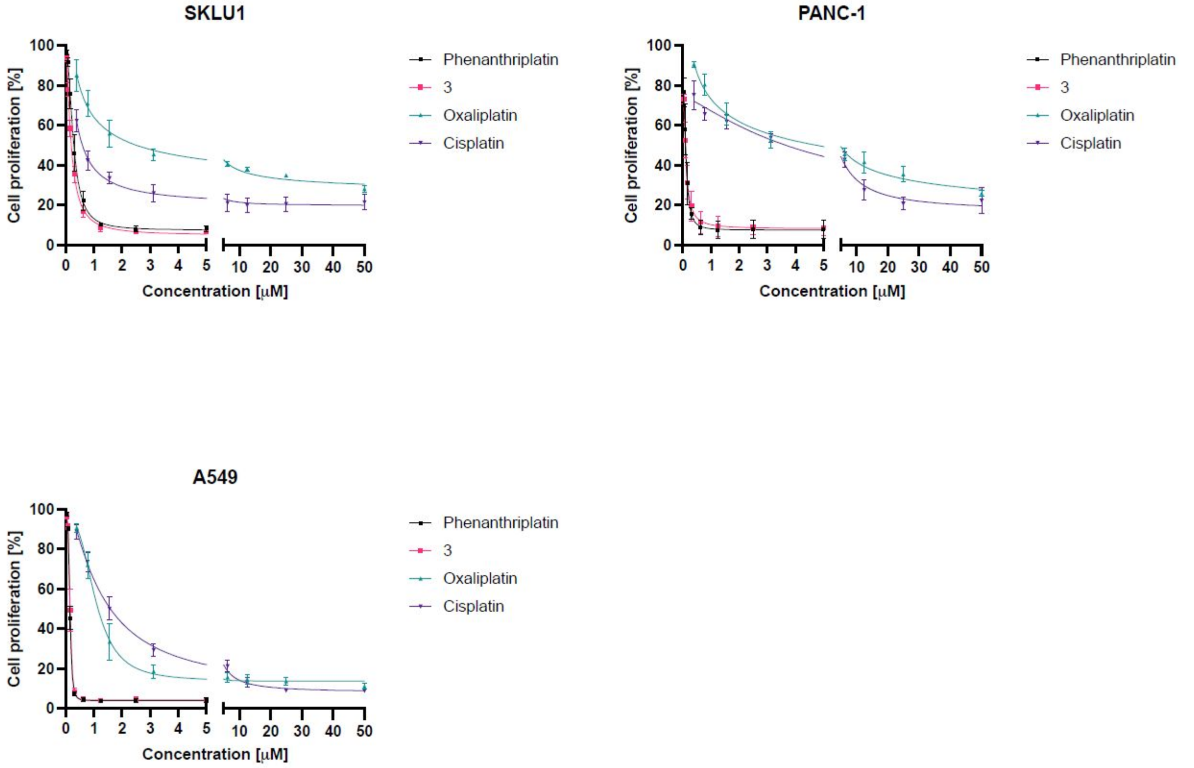

Figure S2. *In-vitro* proliferation of A549, SK-LU-1 and PANC-1 cells following treatment with **3** and Pt-controls for 6 days.

Synthesis & Characterization of Ligands:

****Note*: While no issues were encountered during our handling of the organic azides and **1, 2** and **3**, organic and inorganic azides are known to be neurotoxic and explosive in nature. As such, care should be taken if preparing the compounds described below.*

Complete synthetic route for the synthesis of complexes **1**-**3**:

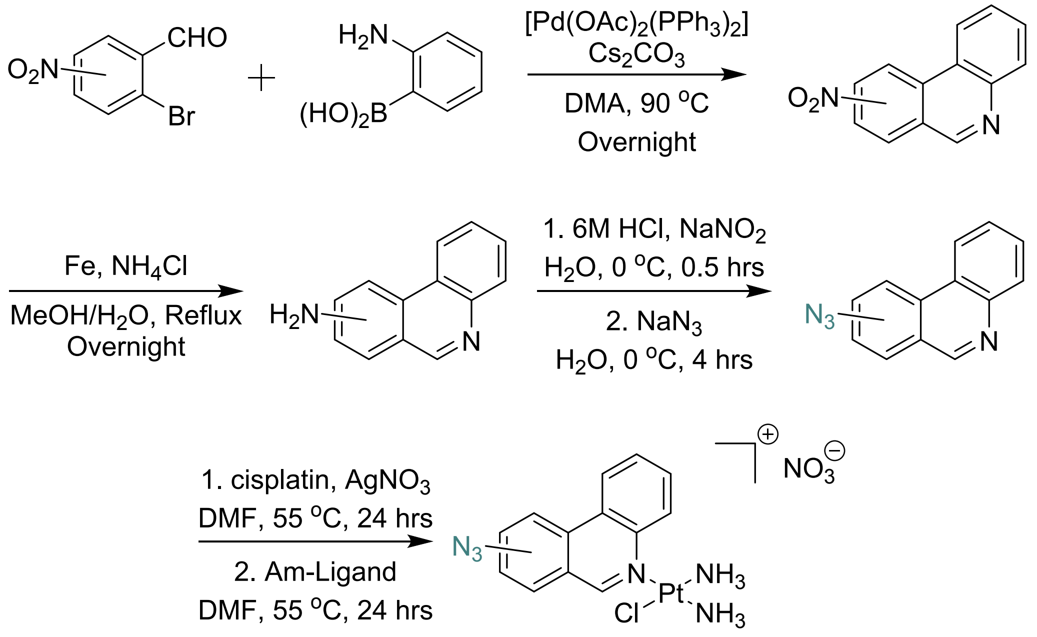

1-3

General Suzuki Coupling Procedure:

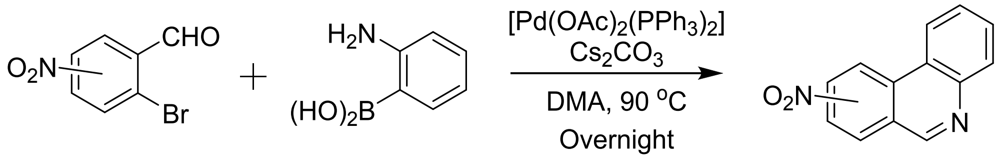

2-bromo-*n*-nitrobenzaldehyde (1.50 g, 6.52 mmol), (2-aminophenyl)boronic acid (1.07 g, 7.83 mmol) and cesium carbonate (3.19 g, 9.78 mmol) were dissolved in DMA (10 mL). To this was added a mixture of [Pd(OAc)$_2$] (73.2 mg, 0.33 mmol) and triphenylphosphine (427.6 mg, 1.63 mmol) in DMA (5 mL). The resulting mixture was heated to 90 ^{o}C and stirred under Ar for 2 days. After stirring, the reaction mixture was cooled to room temperature and diluted with water (50 mL). The product was then extracted with ethyl acetate (3 x 50 mL), washed with brine (3 x 50 mL) and dried over anhydrous sodium sulphate. The solvent was removed under reduced pressure and the product purified by column chromatography (70: 30 petroleum ether: ethyl acetate).

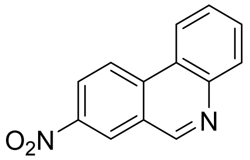

8-nitrophenanthridine was isolated as a brown solid, (693 mg, 47%). ^{1}H NMR (400 MHz, $CDCl_3$) δ 9.39 (s, 1H), 8.94 (d, 1H, *J* = 2.2 Hz), 8.73 (d, 1H, *J* = 9.1 Hz), 8.65 – 8.57 (m, 2H), 8.28 – 8.22 (m, 1H), 7.91 – 7.84 (m, 1H), 7.82 – 7.74 (m, 1H). ^{13}C NMR (101 MHz, $CDCl_3$) δ 153.28, 146.47, 145.78, 136.53, 130.99, 130.76, 128.31, 125.67, 124.75, 124.65, 123.89, 123.14, 122.94. MS (ESI+) $[M+H]^+$: m/z calcd for $C_{13}H_9N_2O_2$: 225.1, found: 224.8.

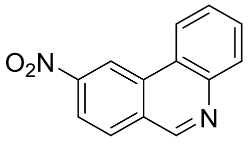

9-nitrophenantrhidine was isolated as a brown solid, (392 mg, 27%). ^{1}H NMR (400 MHz, $CDCl_3$) δ 9.49 (d, 1H, *J* = 1.4 Hz) , 9.39 (s, 1H), 8.64 (d, 1H, *J* = 8.1 Hz), 8.49 (dd, 1H, *J* = 8.7, 2.0 Hz), 8.26 (d, 1H, *J* = 8.0 Hz), 8.22 (d, 1H, *J* = 8.7 Hz), 7.86 (m, 1H), 7.81 (m, 1H). ^{13}C NMR (101 MHz, $CDCl_3$) δ 152.44, 148.96, 145.03, 132.87, 130.82, 130.48, 130.34, 128.74, 128.52, 123.67, 122.57, 121.48, 118.44. (ESI+) $[M+H]^+$: m/z calcd for $C_{13}H_9N_2O_2$: 225.1, found: 224.7.

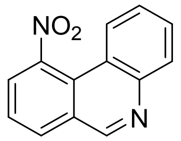

10-nitrophenanthridine was isolated as a red solid, (964 mg, 66%). ^{1}H NMR (400 MHz, $CDCl_3$) δ 9.31 (s, 1H), 8.27 – 8.19 (m, 2H), 8.09 (dd, 1H, *J* = 8.5, 0.7 Hz), 7.95 (dd, 1H, *J* = 7.6, 1.2 Hz), 7.85 – 7.73 (m, 2H), 7.64 (m, 1H). ^{13}C NMR (101 MHz, $CDCl_3$) δ 152.45, 147.74, 145.71, 132.34, 131.04, 130.36, 128.09, 127.73, 127.04, 126.15, 124.15, 123.53, 119.64. (ESI+) $[M+H]^+$: m/z calcd for $C_{13}H_9N_2O_2$: 225.1, found: 225.1.

General Procedure for Reduction of Nitro-group:

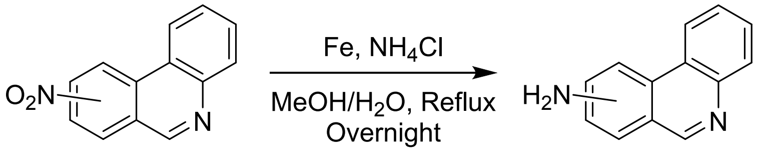

n-nitrophenanthridine (1.00 g, 4.46 mmol), iron powder (1.99 g, 35.68 mmol), and ammonium chloride (2.39 g, 44.60 mol) were added to a round bottom flask. Methanol (40 mL) and water (5 mL) were added to the mixture and the resulting suspension heated to 75 ^oC. The mixture was stirred under reflux overnight and reaction completion was confirmed by TLC the following day. The reaction mixture was allowed to cool to room temperature and the pH of the solution was adjusted to < 5 using HCl (1 M). The solution was extracted with ethyl acetate (2 x 25 mL) and the combined organic layers discarded. The pH of the aqueous phase was then adjusted to > 10 using NaOH (1 M) and the phase again extracted with ethyl acetate (3 x 50 mL). The organic layers were then combined, washed with brine (2 x 25 mL) and dried over anhydrous sodium sulphate. The solvent was removed under reduced

pressure to give a crude solid which was purified by column chromatography (70: 30 ethyl acetate: petroleum ether).

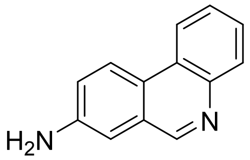

Phenanthridin-8-amine was isolated as a brown solid, (693 mg, 80%). ^1H NMR (400 MHz, $CDCl_3$) δ 9.08 (s, 1H), 8.44 – 8.37 (m, 2H), 8.13 – 8.07 (m, 1H), 7.65 – 7.57 (m, 2H), 7.26 – 7.22 (m, 1H), 7.17 (d, 1H, *J* = 2.3 Hz), 4.11 (s, 2H). ^{13}C NMR (101 MHz, $CDCl_3$) δ 152.87, 146.05, 143.35, 130.13, 128.18, 127.17, 127.13, 125.34, 124.75, 123.46, 121.49, 121.28, 110.59. (ESI+) $[M+H]^+$: m/z calcd for $C_{13}H_{11}N_2$: 195.1, found: 194.9.

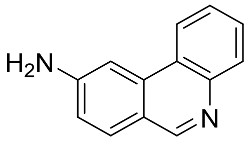

Phenanthridin-9-amine was isolated as a brown solid, (788 mg, 91%). ^1H NMR (400 MHz, $CDCl_3$) δ 9.06 (s, 1H), 8.41 (d, 1H, *J* = 8.1 Hz), 8.10 (d, 1H, *J* = 8.2 Hz), 7.83 (d, 1H, *J* = 8.5 Hz), 7.72 – 7.66 (m, 2H), 7.60 (m, 1H), 7.04 (m, 1H), 4.29 (s, 2H). ^{13}C NMR (101 MHz, $CDCl_3$) δ 152.97, 149.32, 145.05, 134.87, 130.76, 130.00, 128.67, 126.26, 123.79, 122.37, 120.30, 117.31, 103.80. (ESI+) $[M+H]^+$: m/z calcd for $C_{13}H_{11}N_2$: 195.1, found: 194.8.

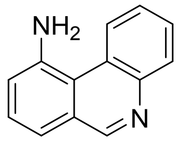

Phenanthridin-10-amine was isolated as a brown solid, (768 mg, 89%). ^1H NMR (400 MHz, $CDCl_3$) δ 9.16 (s, 1H), 9.05 (dd, *J* = 8.3, 1.0 Hz, 1H), 8.20 (dd, *J* = 8.0, 1.3 Hz, 1H), 7.73 – 7.67 (m, 1H), 7.67 – 7.61 (m, 1H), 7.55 – 7.49 (m, 2H), 7.18 (dd, *J* = 6.2, 2.6 Hz, 1H). ^{13}C NMR (101 MHz, $CDCl_3$) δ 154.51, 145.07, 144.61, 130.45, 128.71, 127.87, 127.64, 126.79, 124.97, 121.34, 120.58, 119.42. (ESI+) $[M+H]^+$: m/z calcd for $C_{13}H_{11}N_2$: 195.1, found: 195.1.

General Procedure for Nitro to Azide Transformation:

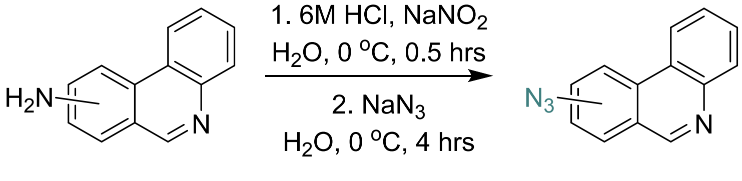

6M HCl (3.9 mL, 23.4 mmol) was added to phenanthridin-*n*-amine (0.75g, 3.86 mmol) and the resulting mixture cooled to 0 °C in an ice bath. Sodium nitrite (0.525 g, 7.623 mmol) in water (5 mL) was added dropwise to this mixture with vigorous stirring, and the resulting solution stirred for 30 minutes at 0 °C. Following this a solution of sodium azide (0.99 g, 15.24 mmol) in water (5 mL) was added dropwise

and the reaction mixture stirred for a further 4 hour at 0 °C. Following reaction completion, the pH of the solution was adjusted to > 7 with sodium hydroxide (1 M) and the product extracted with ethyl acetate (3 x 50 mL). The organic layers were combined, washed with brine (2 x 25 mL) and dried over anhydrous sodium sulphate. The solvent was removed under reduced pressure and the crude product purified by column chromatography (50: 50 petroleum ether: ethyl acetate).

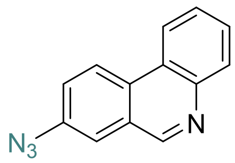

8-azidophenanthridine was isolated as a white solid, (544 mg, 64%). ^{1}H NMR (400 MHz, DMSO) δ 9.48 (s, 1H), 8.88 (d, 1H, *J* = 8.9 Hz), 8.84 – 8.77 (m, 1H), 8.20 – 8.13 (m, 1H), 8.06 (d, 1H, *J* = 2.3 Hz), 7.81 (m, 2H), 7.72 (dd, 1H, *J* = 8.8, 2.4 Hz). ^{13}C NMR (101 MHz, DMSO) δ 152.13, 141.36, 139.52, 129.45, 129.20, 128.15, 128.03, 126.55, 124.76, 124.58, 123.61, 123.02, 118.04. (ESI+) $[M-N_2]^+$: m/z calcd for $C_{13}H_8N_2$: 192.1, found: 192.6.

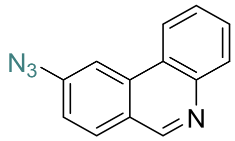

9-azidophenanthridine was isolated as a white solid, (485 mg, 57%). ^{1}H NMR (400 MHz, $CDCl_3$) δ 9.21 (s, 1H), 8.48 (d, 1H, *J* = 8.0 Hz), 8.18 (d, 1H, *J* = 8.0 Hz), 8.13 (d, 1H, *J* = 1.6 Hz), 8.03 (d, 1H, *J* = 8.5 Hz), 7.81 – 7.73 (m, 1H), 7.73 – 7.64 (m, 1H), 7.36 (dd, 1H, *J* = 8.5, 2.1 Hz). ^{13}C NMR (101 MHz, $CDCl_3$) δ 152.82, 144.94, 143.02, 134.12, 130.93, 130.38, 129.45, 127.30, 123.87, 123.39, 122.38, 119.53, 111.06. (ESI+) $[M-N_2]^+$: m/z calcd for $C_{13}H_8N_2$: 192.1, found: 192.3.

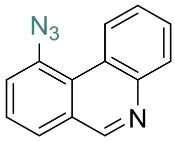

10-azidophenanthridine was isolated as a white solid, (655 mg, 77%). ^{1}H NMR (400 MHz, $CDCl_3$) δ 9.65 (dd, 1H, *J* = 8.4, 1.0 Hz), 9.21 (s, 1H), 8.19 (dd, 1H, *J* = 8.1, 1.2 Hz), 7.84 (dd, 1H, *J* = 7.6, 1.1 Hz), 7.79 – 7.63 (m, 4H). ^{13}C NMR (101 MHz, $CDCl_3$) δ 153.47, 145.35, 137.06, 130.28, 128.81, 128.68, 127.58, 127.39, 127.12, 126.01, 124.36, 123.32, 121.31. (ESI+) $[M-N_2]^+$: m/z calcd for $C_{13}H_8N_2$: 192.1, found: 193.1.

SI NMR (^{1}H & ^{13}C) and HRMS Spectra for 1, 2 and 3:

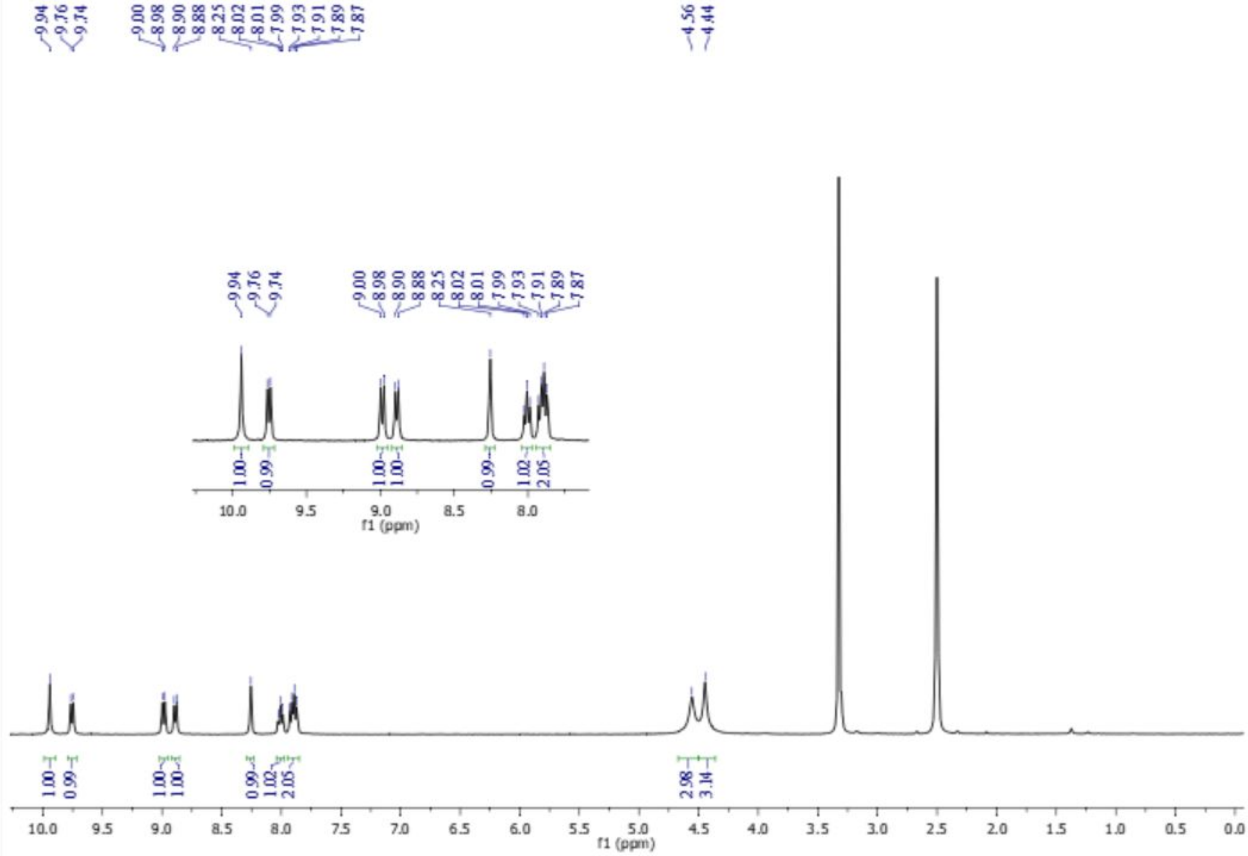

Figure S3. ^{1}H NMR of **1** in DMSO-d_6

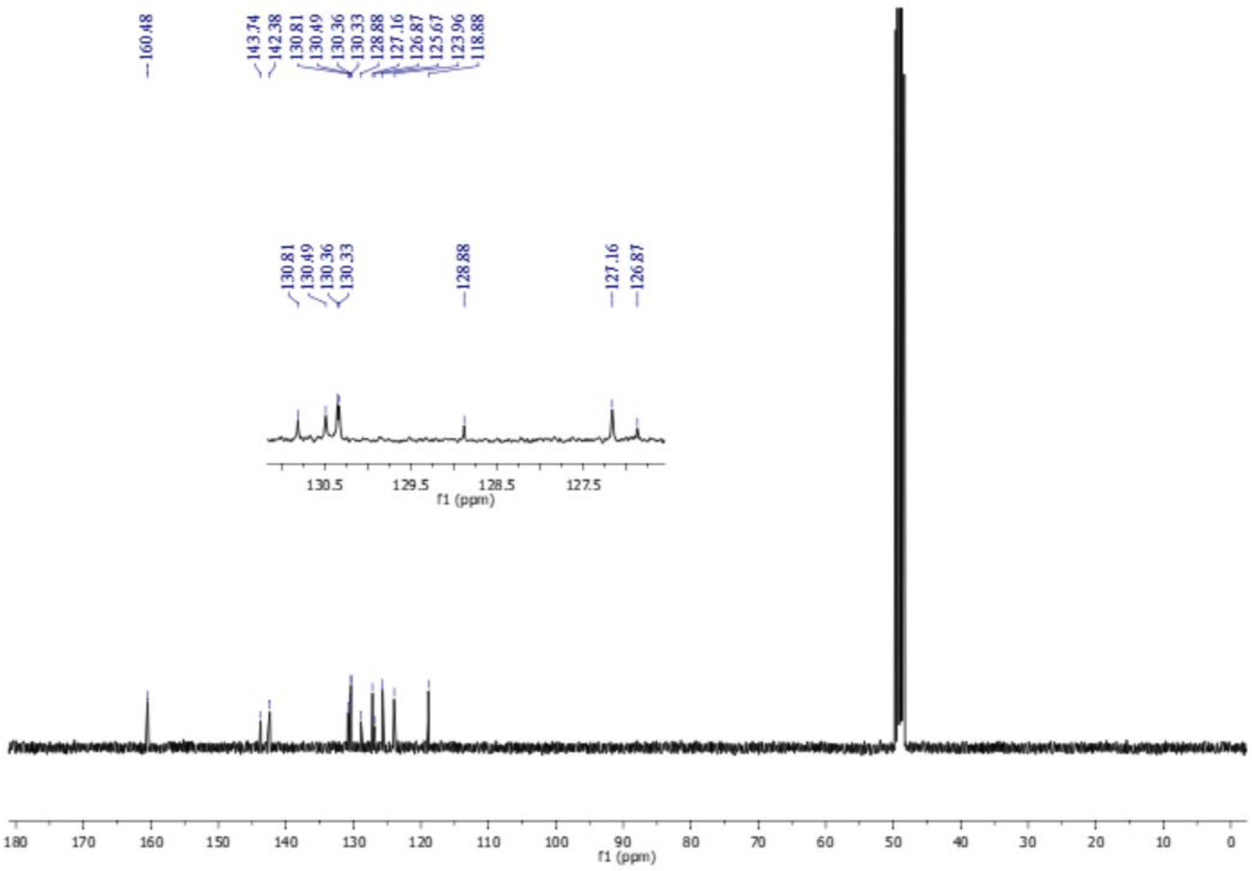

Figure S4. ^{13}C NMR of **1** in MeOD-d_4

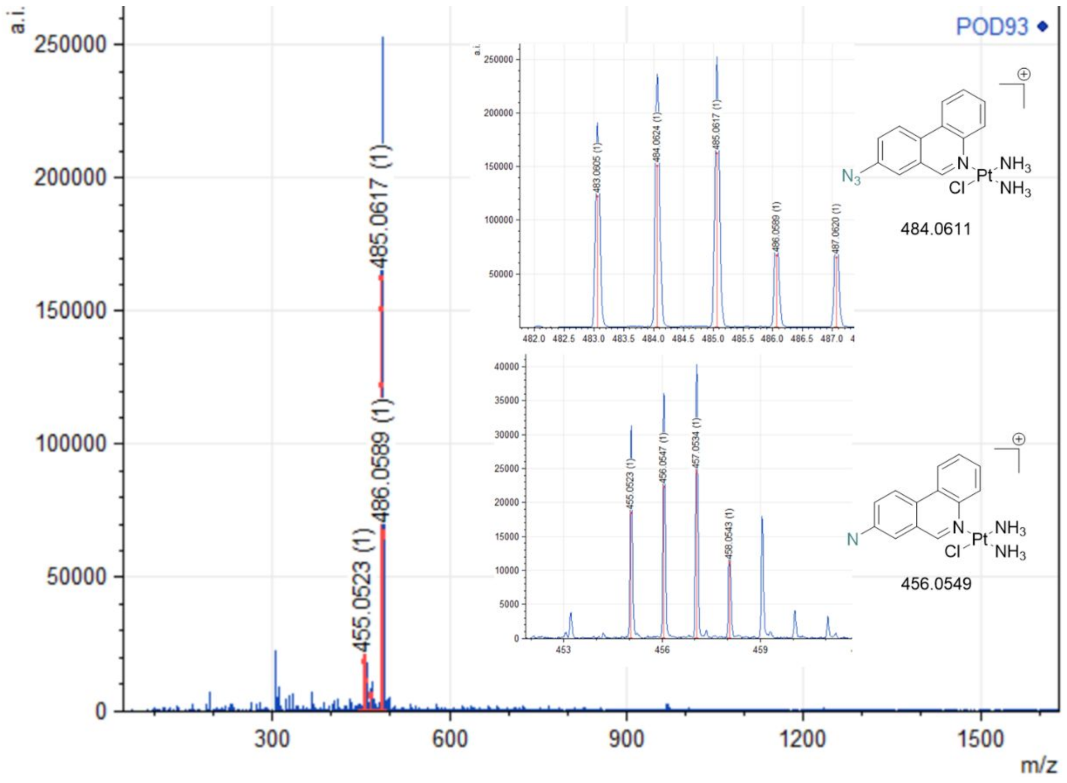

Figure S5. HRMS (ESI+) of **1**

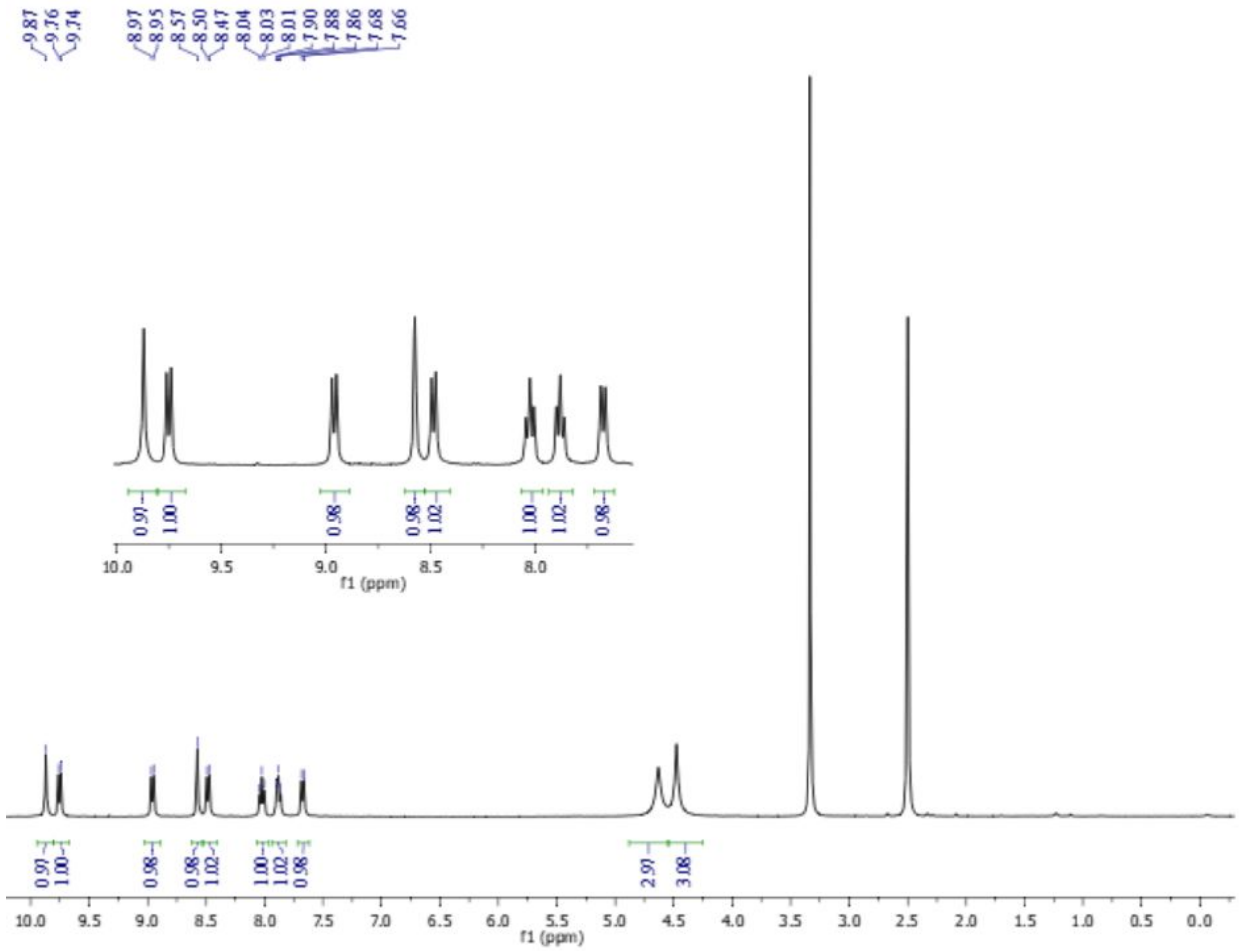

Figure S6. ^{1}H NMR of **2** in DMSO-d_6

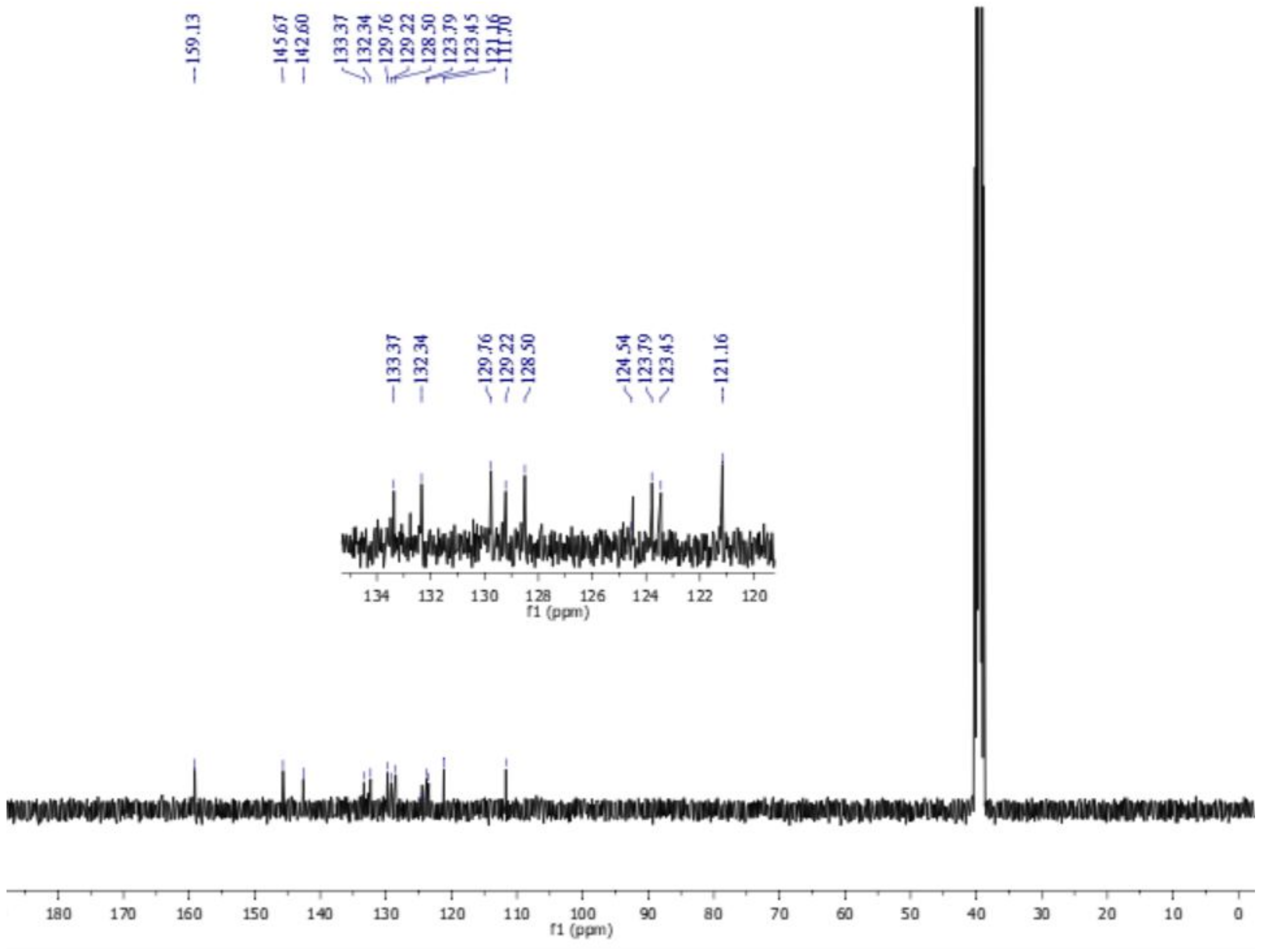

Figure S7. ^{13}C NMR of **2** in DMSO-d_6

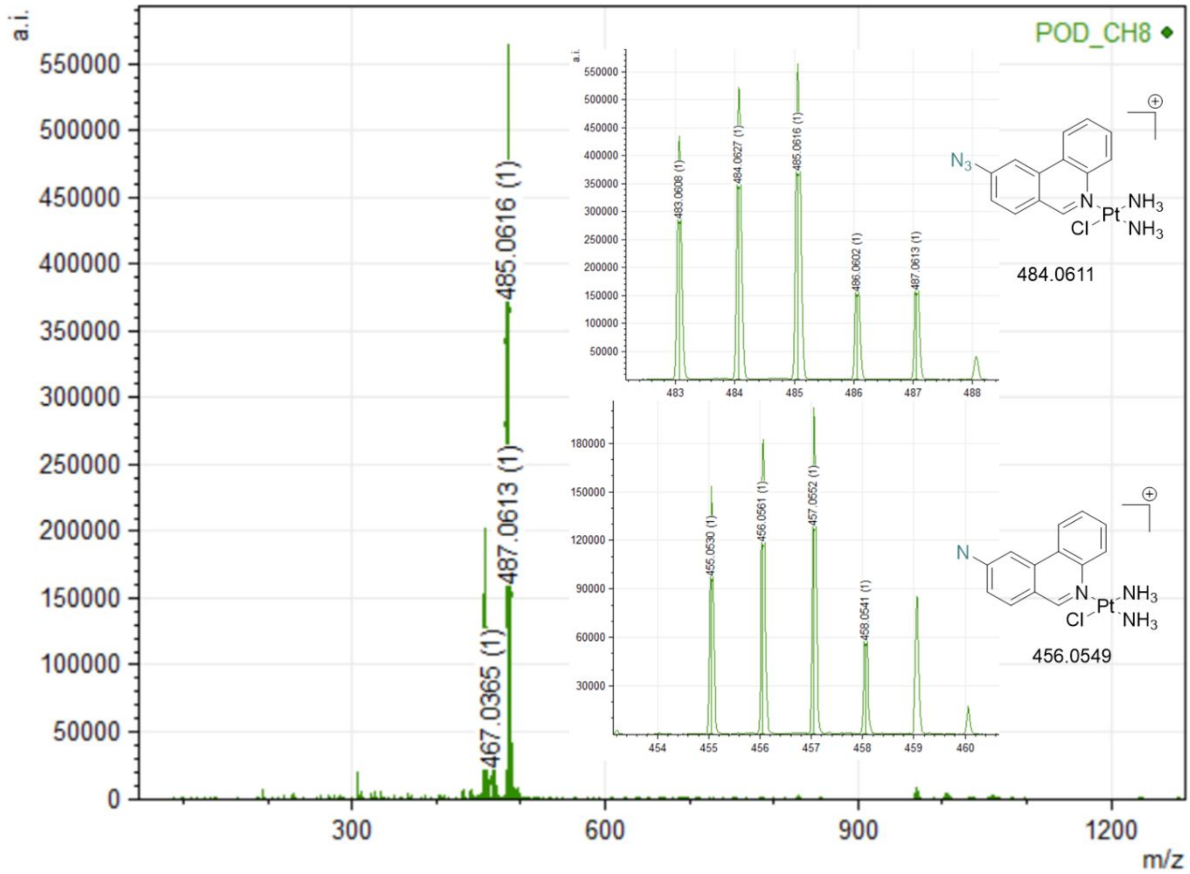

Figure S8. HRMS (ESI+) of **2**

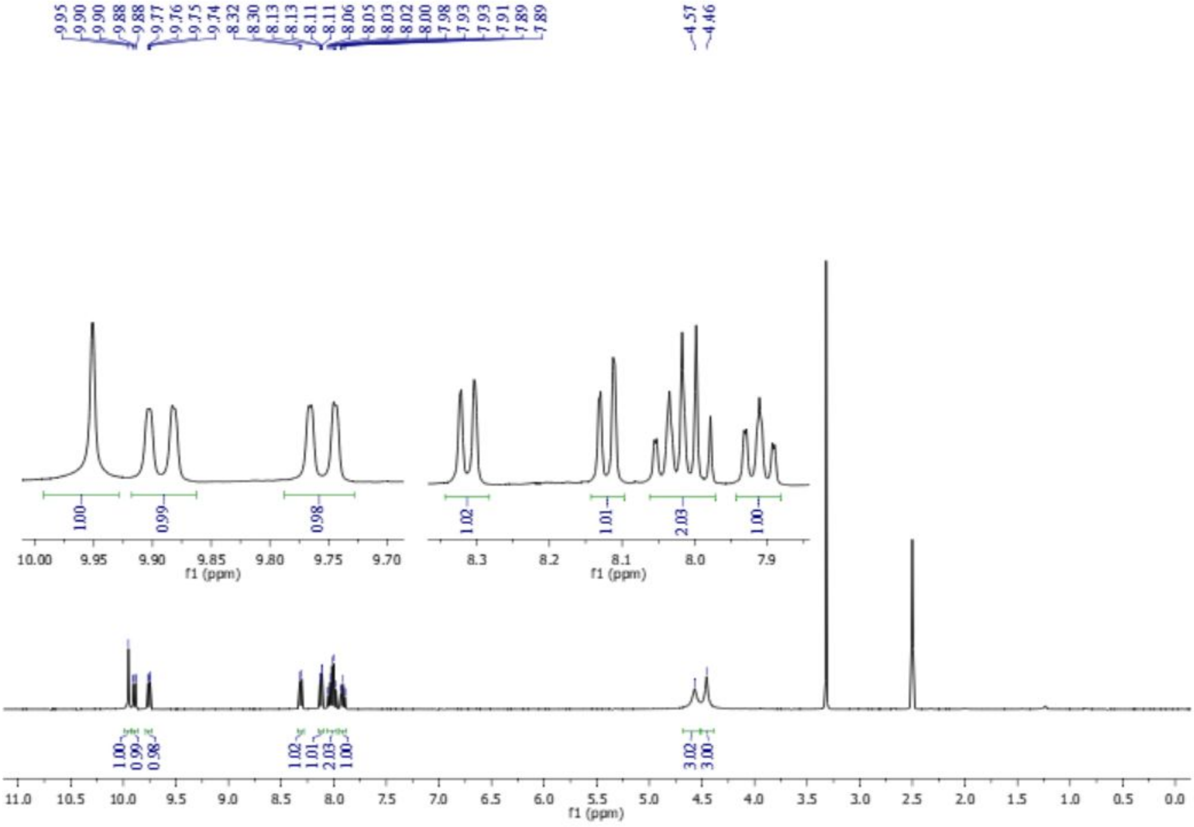

Figure S9. ^{1}H NMR of **3** in DMSO-d_6

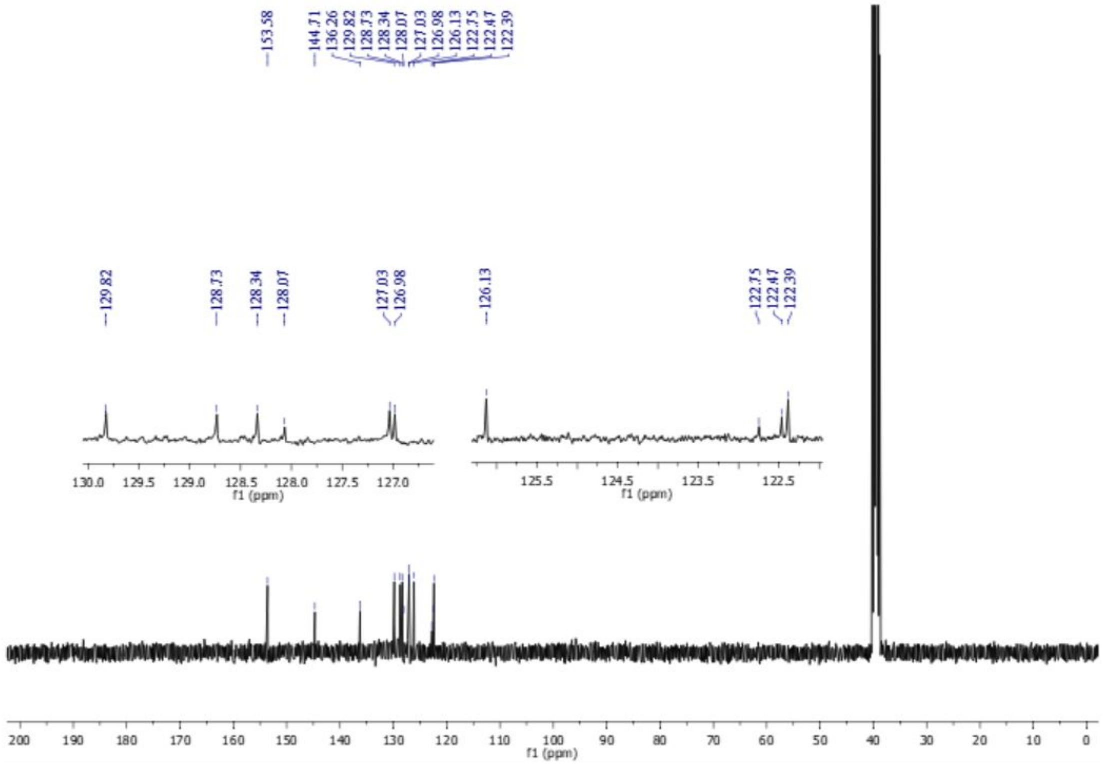

Figure S10. ^{13}C NMR of **3** in DMSO-d_6

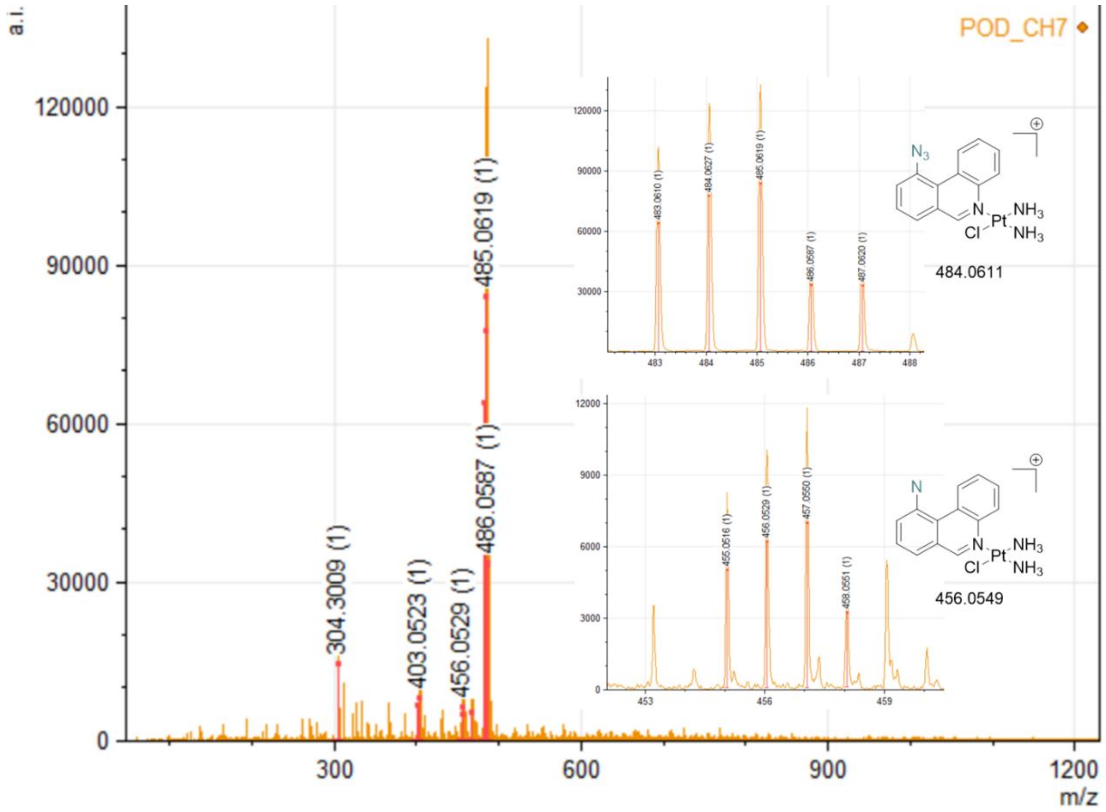

Figure S11. HRMS (ESI+) of **3**

Single crystal X-Ray diffraction:

The X-ray intensity data for **1** were measured (λ = 1.54178 Å) on a Bruker Apex Kappa Duo with an Oxford Cobra Cryosystem low temperature device at 100(2) K using a MiTeGen micromount and NVH immersion oil. See Table S1 for crystal data and structure refinement details.

Bruker APEX[1] software was used to collect and reduce data and correct for Lorentz and polarization effects. Data were corrected for absorption effects using the Multi-Scan method SADABS.[2] Structures were solved with the SHELXT[3] structure solution program using Intrinsic Phasing and refined using Least Squares method on F^2 with SHELXL[4] within the OLEX2[5] package. All non-hydrogen atoms were refined anisotropically. Hydrogen atoms were placed in calculated positions with Uiso dependencies derived from their carrier atoms (riding model).

In **1** the azide group is disordered and modelled over two locations (75:25% occupancy) and refined using geometric (SADI, DFIX) and displacement (SIMU, ISOR) restraints. The methanol OH hydrogen was located on the difference map and refined using geometric restraints (DFIX).

Crystallographic data have been deposited with the Cambridge Crystallographic Data Centre as supplementary publication no. 2296496. Copies of the data can be obtained, free of charge, on application to CCDC, 12 Union Road, Cambridge CB2 1EZ, UK, (fax: +44-(0)1223-336033 or e-mail:deposit@ccdc.cam.ac.uk).

Table S1. Crystal data and structure refinement for **1**

Identification code	TCD1950
CCDC No.	2296496
Empirical formula	$C_{14}H_{18}ClN_7O_4Pt$
Formula weight	578.89
Temperature (K)	100(2)
Crystal system	Monoclinic
Space group	$P2_1/n$
a (Å)	14.8299(10)
b (Å)	7.2741(5)
c (Å)	17.2890(12)
α (°)	90
β (°)	96.980(3)
γ (°)	90
Volume (Å^3)	1851.2(2)

[1] Bruker (2017). APEX3, Bruker AXS Inc., Madison, WI, USA.
[2] SADABS: Krause, L., Herbst-Irmer, R., Sheldrick, G. M., Stalke, D. (2015). *J. Appl. Cryst.* 48, 3-10.
[3] Sheldrick, G. M. (2015). *Acta Cryst.* A71, 3-8
[4] Sheldrick, G. M. (2015). *Acta Cryst.* C71, 3-8.
[5] OLEX2: Dolomanov, O.V., Bourhis, L.J., Gildea, R.J, Howard, J.A.K. & Puschmann, H. (2009), *J. Appl. Cryst.* 42, 339-341.

Z	4
ρcalcg (cm^3)	2.077
μ (mm-1)	15.846
F(000)	1112.0
Crystal size (mm^3)	0.106 × 0.074 × 0.018
Radiation	Cu Kα (λ = 1.54178)
2Θ range for data collection (°)	7.422 to 140.21
Reflections collected	21407
Independent reflections	3482<br>R_{int} = 0.0602<br>R_{sigma} = 0.0485
Data/restraints/parameters	3482/89/278
Goodness-of-fit on F^2	1.141
Final R indexes [I≥2σ (I)]	R_1 = 0.0322,<br>wR_2 = 0.0835
Final R indexes [all data]	R_1 = 0.0394,<br>wR_2 = 0.0873
Largest diff. peak/hole (e Å^{-3})	1.07/-1.16

Table S2. Crystal data and structure refinement for **1**.

Identification code	TCD1950
CCDC No.	2296496
Empirical formula	$C_{14}H_{18}ClN_7O_4Pt$
Formula weight	578.89
Temperature (K)	100(2)
Crystal system	Monoclinic
Space group	$P2_1/n$
a (Å)	14.8299(10)
b (Å)	7.2741(5)
c (Å)	17.2890(12)
α (°)	90
β (°)	96.980(3)
γ (°)	90
Volume (Å^3)	1851.2(2)
Z	4
ρcalcg (cm^3)	2.077
μ (mm-1)	15.846
F(000)	1112.0
Crystal size (mm^3)	0.106 × 0.074 × 0.018
Radiation	Cu Kα (λ = 1.54178)
2Θ range for data collection (°)	7.422 to 140.21
Reflections collected	21407
Independent reflections	3482<br>R_{int} = 0.0602<br>R_{sigma} = 0.0485
Data/restraints/parameters	3482/89/278
Goodness-of-fit on F^2	1.141
Final R indexes [I≥2σ (I)]	R_1 = 0.0322,<br>wR_2 = 0.0835
Final R indexes [all data]	R_1 = 0.0394,<br>wR_2 = 0.0873
Largest diff. peak/hole (e Å^{-3})	1.07/-1.16

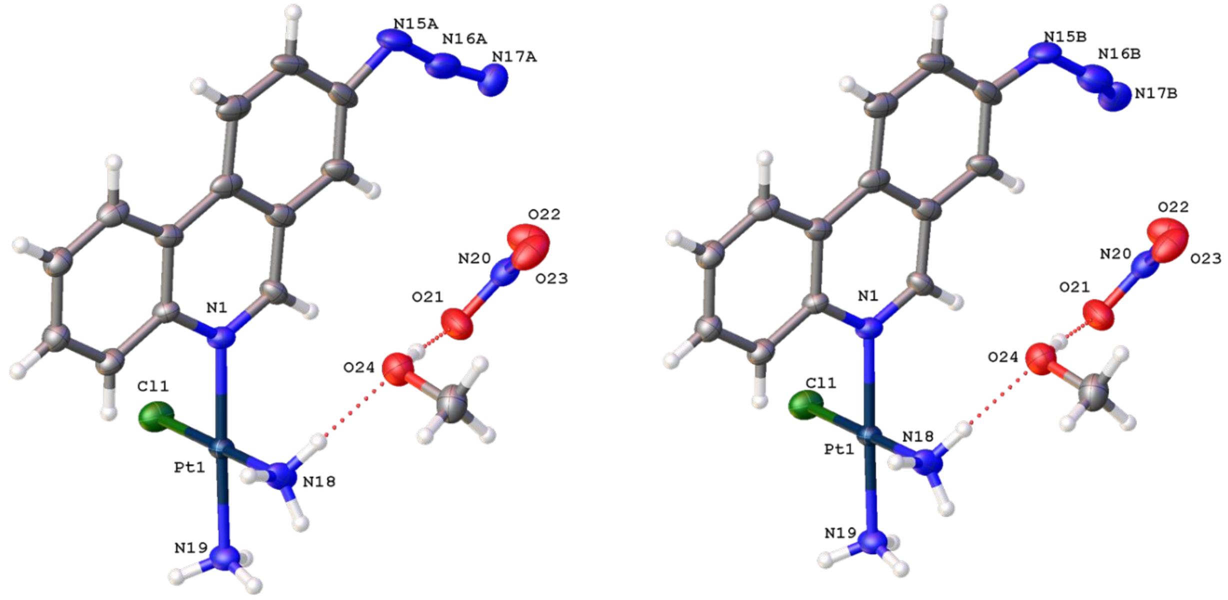

Figure S12. Individual images of each disordered moiety of **1** with (A) 75% occupied and (B) 25% occupied. Atomic displacement shown at 50% probability and heteroatoms labelled only.

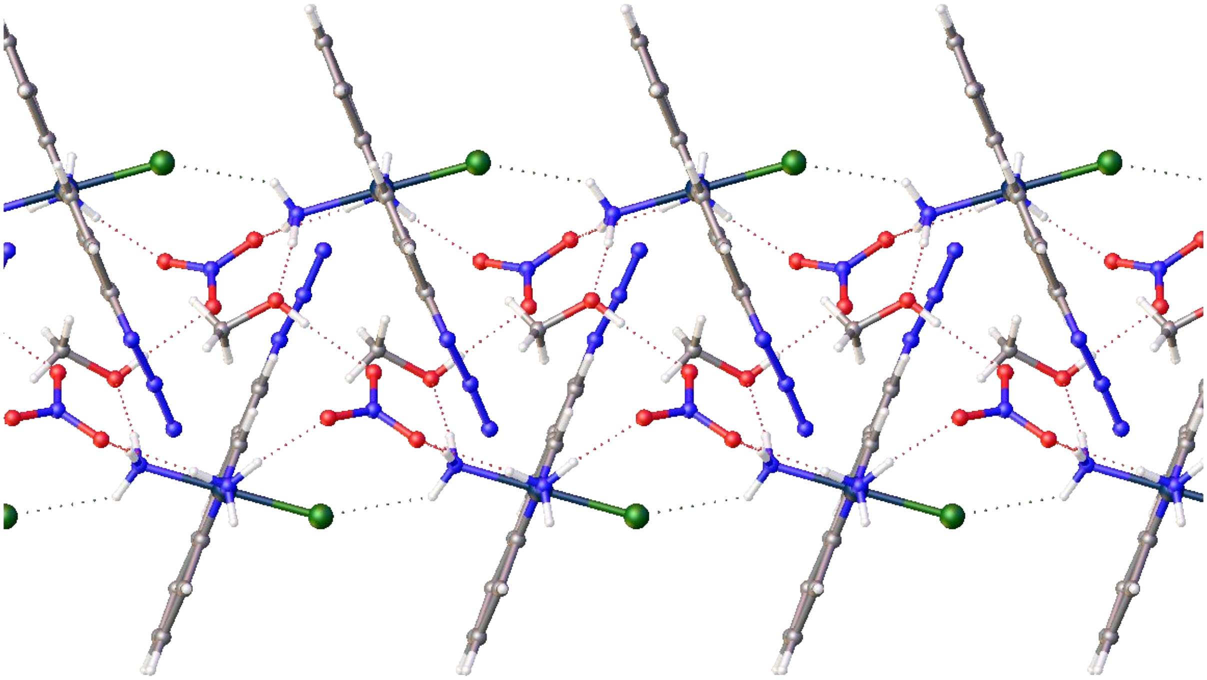

Figure S13. Strong hydrogen bonding network in **1** viewed normal to the c-axis. Hydrogen bonding represented by dotted lines. Atomic displacement shown at 50% probability.

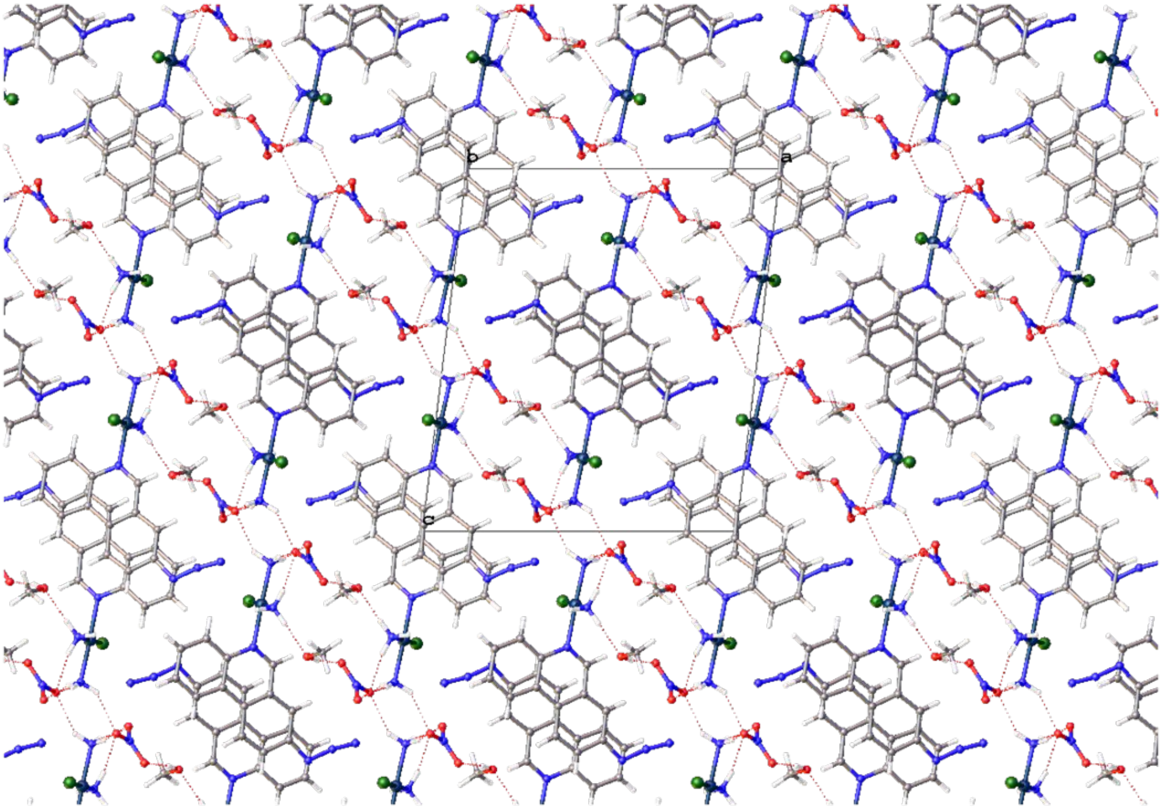

Figure S14. Schematic packing diagram of the major occupied moiety in **1** viewed normal to the b-axis. Dotted lines indicate hydrogen bonding interactions.

Table S3. Hydrogen Bonds for **1**.

D	H	A	d(D-H)/Å	d(H-A)/Å	d(D-A)/Å	D-H-A/°
N18	H18A	O24	0.91	1.96	2.862(6)	174
N18	H18B	O23[1]	0.91	2.08	2.978(6)	167
N19	H19A	O22[2]	0.91	2.10	2.999(6)	171
N19	H19B	O23[3]	0.91	2.38	3.087(7)	134
N19	H19C	O23[1]	0.91	2.29	3.192(6)	169
O24	H24	O21	0.842(10)	1.959(16)	2.796(6)	172(7)

[1]1/2-X,-1/2+Y,3/2-Z; [2]1/2-X,1/2+Y,3/2-Z; [3]1/2+X,3/2-Y,1/2+Z

Table S4. Bond lengths (Å) and Angles (°) for **1**, phenanthriplatin and the triflate derivative

	**1**	**phenanthriplatin**	**triflate**
Pt1-Cl1	2.2946(14)	2.2998(19)	2.2962(7)
Pt1-N1	2.045(5)	2.032(6)	2.047(2)
Pt1-N18	2.043(4)	2.040(6)	2.025(2)
Pt1-N19	2.047(5)	2.036(6)	2.039(2)
N1-Pt1-Cl1	91.70(12)	85.90(18)	87.72(6)
N1-Pt1-N19	178.28(18)	176.4(2)	177.97(9)
N18-Pt1-Cl1	179.31(15)	178.26(19)	179.31(6)
N18-Pt1-N1	88.58(17)	94.2(3)	92.96(9)
N18-Pt1-N19	91.57(18)	89.4(3)	89.05(9)
N19-Pt1-Cl1	88.13(13)	90.51(19)	90.27(6)

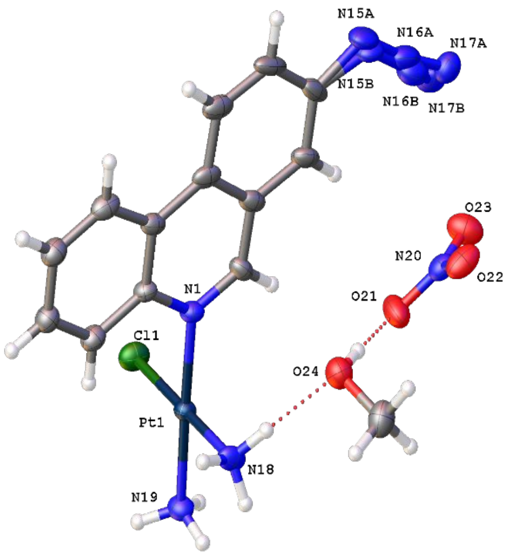

Figure S15. Disordered molecular structure of 1, showing the NO_3^- counter ion and MeOH solvate. Atomic displacement shown at 50% probability and heteroatoms labelled only.

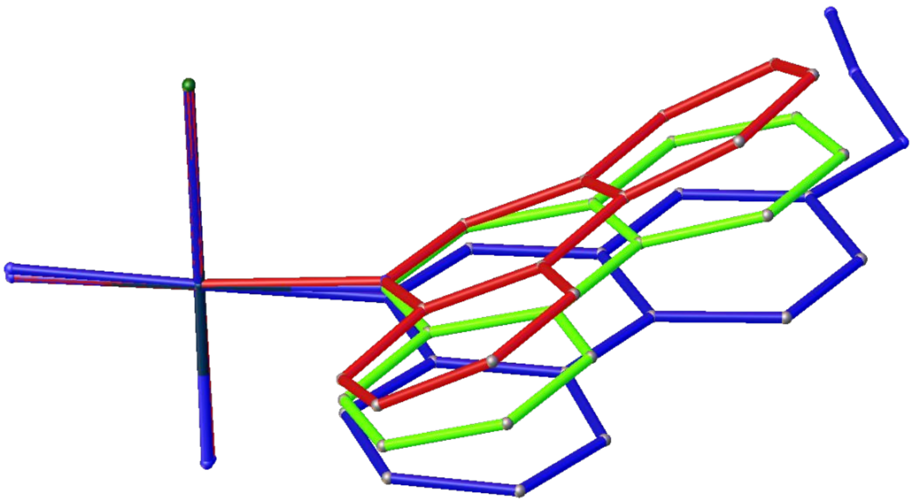

Figure S16. Overlay of **1** (Blue), triflate salt of phenanthriplatin (Green) and nitrate salt of phenanthriplatin (Red).

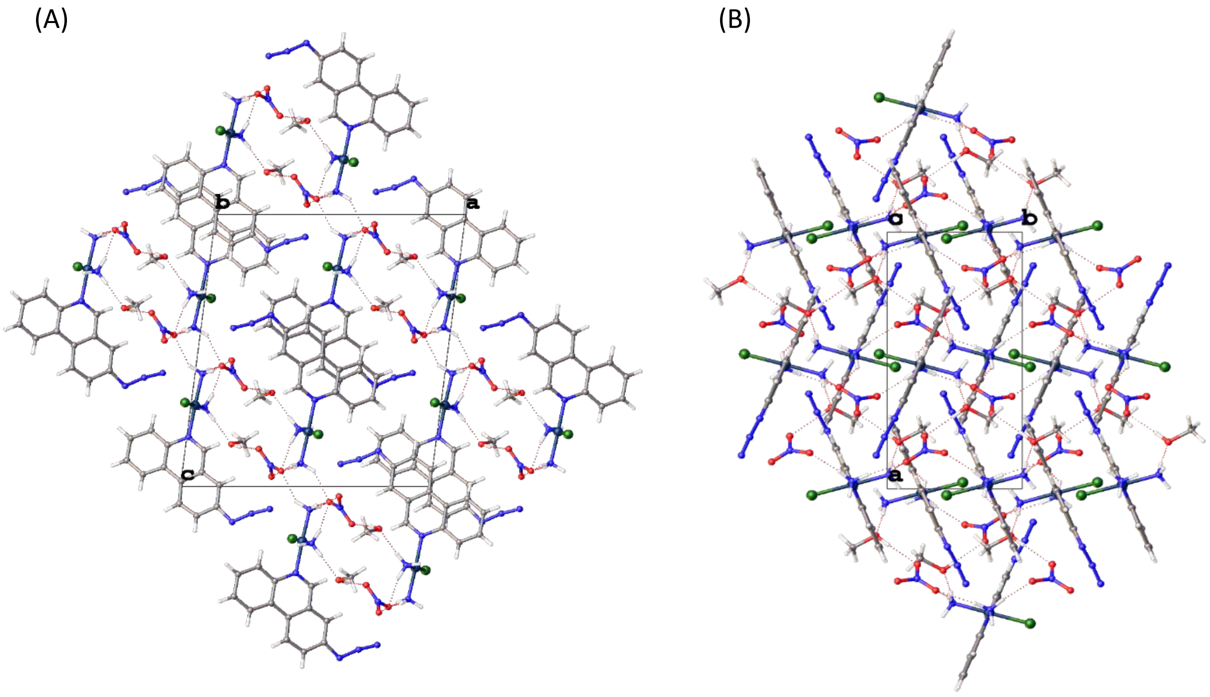

Figure S17. Schematic packing diagrams of **1** showing the π-π stacking viewed (A) normal to the B-axis and (B) normal to the c-axis. Dotted lines indicate hydrogen bonding interactions.
